# Tuberculosis Visualized With the Ultrasound Probe: A Systematic Review of Sonographic Pattern Descriptions and an Analysis of Common Sonographic Features

**DOI:** 10.1093/ofid/ofaf010

**Published:** 2025-03-07

**Authors:** Stefan Fabian Weber, Katharina Manten, Katharina Kleiber, Lisa Ruby, Maurizio Grilli, Frank Tobian, Sabine Bélard, Claudia M Denkinger

**Affiliations:** Department of Infectious Diseases and Tropical Medicine, University Hospital Heidelberg, Heidelberg, Germany; Department of Parasitology, University Hospital Heidelberg, Heidelberg, Germany; DZIF Partner Site Heidelberg, German Center for Infectious Disease Research, Heidelberg, Germany; DZIF Partner Site Heidelberg, German Center for Infectious Disease Research, Heidelberg, Germany; Department of Anesthesiology, University Hospital Heidelberg, Heidelberg, Germany; Internal Medicine, Evangelisches Krankenhaus Köln-Kalk, Cologne, Germany; Institute of Tropical Medicine, University of Tübingen, Tübingen, Germany; DZIF Partner Site Tübingen, German Center for Infectious Disease Research, Tübingen, Germany; Library of the Medical Faculty Mannheim of the University of Heidelberg, University Medical Center Mannheim, Mannheim, Germany; Department of Infectious Diseases and Tropical Medicine, University Hospital Heidelberg, Heidelberg, Germany; DZIF Partner Site Heidelberg, German Center for Infectious Disease Research, Heidelberg, Germany; Institute of Tropical Medicine, University of Tübingen, Tübingen, Germany; DZIF Partner Site Tübingen, German Center for Infectious Disease Research, Tübingen, Germany; Department of Infectious Diseases and Tropical Medicine, University Hospital Heidelberg, Heidelberg, Germany; DZIF Partner Site Heidelberg, German Center for Infectious Disease Research, Heidelberg, Germany

**Keywords:** diagnosis, extrapulmonary tuberculosis, pulmonary tuberculosis, tuberculosis, ultrasound

## Abstract

Evidence on tuberculosis (TB) ultrasound patterns is scarce. We systematically reviewed the literature aiming to identify common TB ultrasound features. Sources included PubMed, Cochrane Library, and others (1 January 2000 through 30 August 2021). Any article type (retrospective, prospective, cases, trials) with verbal ultrasound descriptions of TB were included; those with <2 ultrasound features were excluded. We adapted Murad et al (2018) for quality assessment. The outcome was a descriptive frequency ranking of ultrasound features and patterns (combinations) per organ. From 388 publications, 613 ultrasound descriptions across 23 organs from 2167 individuals (465 single cases, 1702 from case series/studies) were extracted. The most commonly described sonographic patterns related to the female breast (n = 45), the liver (n = 40), and the pancreas (n = 37). The synthesis reveals sonographic TB patterns, but is constrained by limited representativeness of studies and the partly subjective analysis. Our review may serve as a clinical or research resource.

**Clinical Trials Registration:**

PROSPERO (CRD42021283319)

## Teaser

This systematic review collated ultrasound features and combinations thereof in tuberculosis disease in various organs; we standardized descriptions for analysis. Representativeness was mostly low, but our review offers a resource for clinical and research purposes and advocates for standardized nomenclature.

Globally, tuberculosis (TB) is the leading single infectious cause of death [1]. Diagnosis of pulmonary TB and extrapulmonary TB relies on chest radiography, cross-sectional imaging (computed tomography, magnetic resonance imaging) in high-affluence settings, and, increasingly, point-of-care ultrasound in limited-resource settings (low- and middle-income countries [LMICs]) [2, 3].

Studies on abdominal and thoracic TB ultrasound typically report on ultrasound findings in a binary way (eg, presence or absence of lymph nodes) [4, 5] rather than describing detailed sonographic patterns. Recognition of sonographic features and patterns is needed to inform the design of diagnostic TB protocols. For example, hypoechoic splenic micro-abscesses have been described for decades [6] but were only later clinically recognized to be characteristic for TB. This recognition led to their incorporation into a defined protocol, focused assessment with sonography for human immunodeficiency virus–related tuberculosis (FASH) [7], which has since been validated prospectively for its diagnostic accuracy.

The objective of this systematic review was to compile and categorize the ultrasound features of TB disease across various organs and to identify common patterns of these features.

## METHODS

### Search Strategy

We developed a search strategy to identify relevant literature in PubMed, Cochrane Library, Web of Science Core Collection, Cumulative Index to Nursing and Allied Health Literature, ClinicalTrials.gov, and the World Health Organization International Clinical Trials Registry Platform using terms relating to “tuberculosis,” “ultrasound,” and “diagnosis” with the help of a librarian (M. G.) (for the search protocol, see PROSPERO identifier CRD42021283319). The search was limited to articles in English, French, Italian, Spanish, or German published from 1 January 2000 till 30 August 2021 to avoid inclusion of older ultrasound systems with inferior imaging capacities. Authors were contacted for unavailable full-text files.

### Study Selection

Inclusion criteria required any publication type (eg, case reports, studies, reviews) to provide original verbal descriptions of TB visualized by ultrasound (extracorporeal or endoscopic) detailing at least 2 features like size, echogenicity, shape, margin, number, and others. Citations from reviews were also assessed for eligibility. Articles were excluded if ultrasound descriptions were not sufficiently detailed (eg, only image provided) or if ultrasound was performed intraoperatively, in nonhuman animals, or after >2 weeks of completed anti-TB treatment.

All identified publications were imported into EndNote 20 software and deduplicated. Title and abstract screening was split in halves and performed separately (K. M. and S. F. W.) after alignment in selection was ensured over the first 50 articles. Unclear publications were discussed and a consensus decision was made.

Full-text review and data extraction (including quality assessment, patient characteristics, and ultrasound description) were split between authors and secondarily reviewed by the other author; disparities were resolved by discussion. Single ultrasound features were extracted from descriptions when they were clear and unambiguous (see Supplementary Table 1 for extracted features).

### Quality Assessment

**Figure 1.**
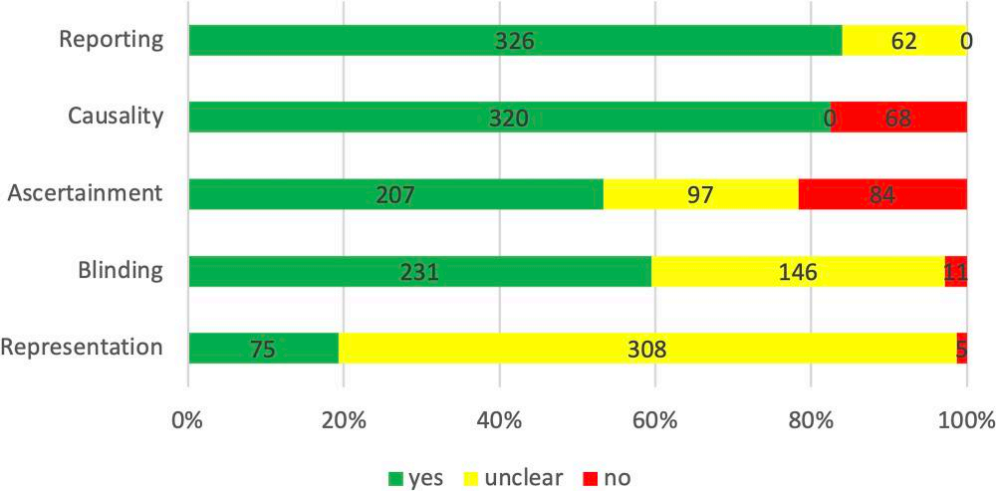
Quality assessment. Reporting: “Is the case(s) description regarding the ultrasound findings sufficient to allow reproduction?” (adequate: 326/388 [84%], unclear or insufficient: 62/388 [16%]). Causality: “Were other alternative causes for ultrasound pathologies ruled out?” (low: 320/388 [82%], high: 68/388 [18%]). Ascertainment: “Was TB adequately ascertained?” (low: 207/388 [53%], unclear: 97/388 [25%], high: 84/388 [22%]). Blinding: “Was the ultrasound operator blinded to the diagnosis?” (low: 231/388 [60%], unclear: 146/388 [38%], not performed: 11/388 [3%]). Representation: “Is the patient(s) representative of the experience of the investigator/center?” (low: 75/388 [19%], uncertain: 308/388 [79%], high: 5/388 [1%]).

Quality assessment was performed using an adapted tool for case reports/series [8]. The domains of representation, blinding, ascertainment, causality, and reporting were covered (predefined, see Prospero Search Protocol, Figure 1). The adapted tool was piloted on 10 publications separately (by S. F. W. and K. M.).

### Analysis

Analyses were done for organs with 8 or more descriptions. Descriptions from each publication were weighted equally regardless of the number of cases reported (eg, single case report vs a pattern in a case series or a study). We computed pairwise and triad combinations of features (R package “combinat”). Within each organ, we then ranked single, pairwise, and triads of sonographic features by frequency. We report the 10 most frequent descriptions or combinations of features. If >1 feature was on the 10th rank or if <2 entries for features or combinations were available, we removed those. Two authors (K. M. and S. F. W.) then summarized these rankings into archetypal descriptions for each organ.

## RESULTS

**Figure 2.**
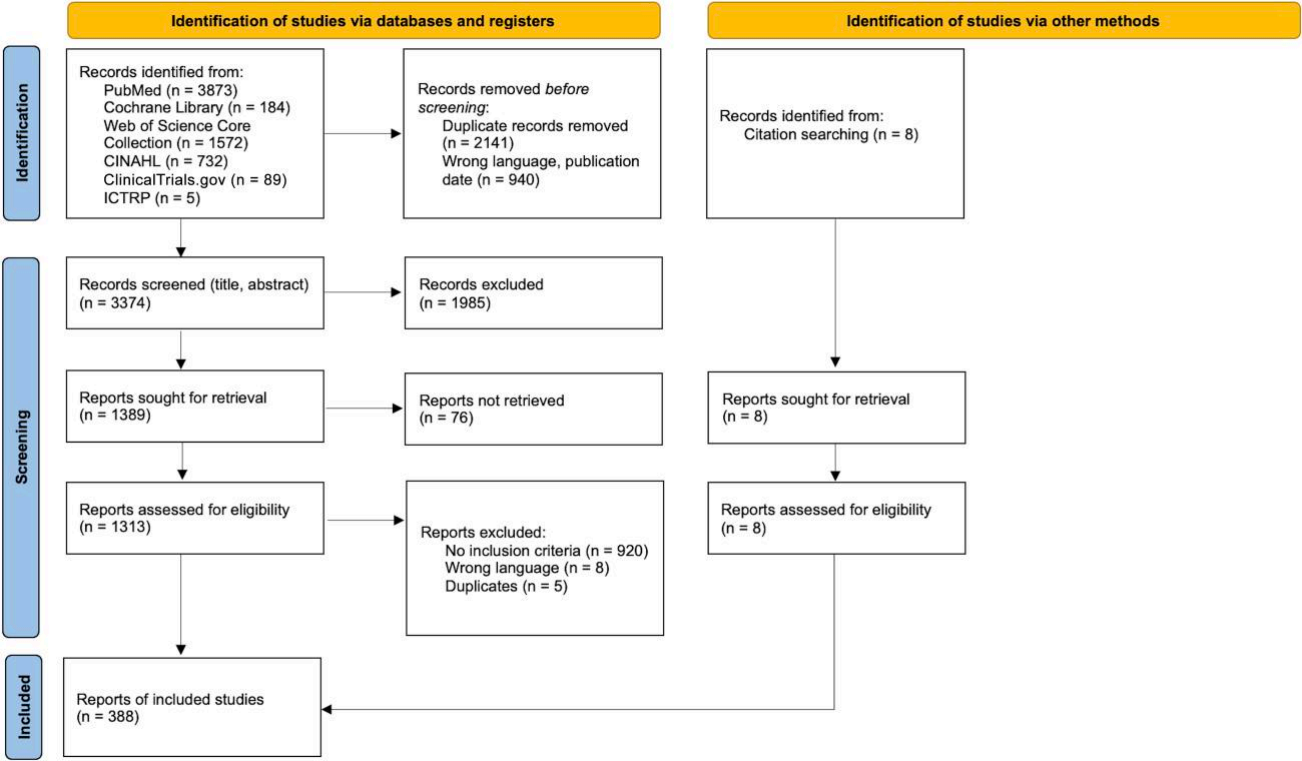
Preferred Reporting Items for Systematic Reviews and Meta-Analyses (PRISMA) flowchart. Abbreviations: CINAHL, Cumulative Index to Nursing and Allied Health Literature; ICTRP, World Health Organization International Clinical Trials Registry Platform.

The search yielded 6455 articles. After de-duplication, language compatibility, and title/abstract and full-text screening as well as identification of publications from reviews, we included 388 articles (Figure 2; see Search Protocol in Supplementary Document 2 and all articles in Supplementary Document 3).

### Quality Assessment

Only 75 of 388 (19%) publications had a low risk for selection bias (representation); risk for ascertainment bias was low in 207 of 388 (53%) articles. For further details, see Figure 1.

### Ultrasound Pattern Descriptions

In the 388 publications, 494 descriptions were based on single cases (>1 per publication possible) and 119 based on case series/studies. Eight or more descriptions were available for 23 organs, with TB affecting the female breast (n = 44), liver (n = 40), and pancreas (n = 37) described most frequently. The least number of descriptions was available for TB affecting the uterus and eyes (n = 8 each). The 5 most frequent sonographic TB features, pair, and triad combinations of features are provided in Table 1 for the 5 most commonly TB-affected organs (see expanded top 10 ranking for all organs in Supplementary Table 2).

**Table 1.** Example Organs: Single Ultrasound Features and Pair and Triad Combinations by Organ and frequency

	Single Feature		Pair (2-Feature) Combinations			Triad (3-Feature) Combinations			
Breast: n = 25 articles; n = 45 descriptions									
1	Number_lesion_: single	n = 28	Number_lesion_: single	Echo_lesion_: mixed	n = 19	Echo_lesion_: mixed	Number_lesion_: single	Margin_lesion_: ill-defined	n = 9
2	Echo_lesion_: mixed	n = 24	Number_lesion_: single	Margin_lesion_: ill-defined	n = 12	Echo_lesion_: mixed	Number_lesion_: single	Post. phen: enh.	n = 7
3	Margin_lesion_: ill-defined	n = 16	Margin_lesion_: ill-defined	Echo_lesion_: mixed	n = 10	Echo_lesion_: mixed	Margin_lesion_: ill-defined	Post. phen: enh.	n = 6
4	Echo_lesion_: hypo	n = 15	Echo_lesion_: hypo	Number_lesion_: single	n = 9	Echo_lesion_: mixed	Number_lesion_: single	Margin_lesion_: well-defined	n = 5
5	Shape_lesion_: round/oval	n = 12	Echo_lesion_: mixed	Post. phen: enh.	n = 9	Echo_lesion_: mixed	Number_lesion_: single	Organ_other_: continuous	n = 5
Liver: n = 30 articles; n = 40 descriptions									
1	Echo_lesion_: hypo	n = 24	Echo_lesion_: hypo	Number_lesion_: multiple	n = 14	Size_lesion_: small	Echo_lesion_: hypo	Number_lesion_: multiple	n = 8
2	Number_lesion_: multiple	n = 21	Echo_lesion_: hypo	Shape_lesion_: round/oval	n = 11	Location_lesion_: dissem.	Echo_lesion_: hypo	Number_lesion_: multiple	n = 8
3	Shape_lesion_: round/oval	n = 17	Location_lesion_: dissem.	Number_lesion_: multiple	n = 10	Size_lesion_: small	Location_lesion_: dissem.	Echo_lesion_: hypo	n = 5
4	Location_lesion_: localized	n = 15	Location_lesion_: localized	Number_lesion_: single	n = 9	Size_lesion_: small	Location_lesion_: dissem.	Number_lesion_: multiple	n = 5
5	Number_lesion_: single	n = 12	Number_lesion_: multiple	Shape_lesion_: round/oval	n = 9	Location_lesion_: dissem.	Number_lesion_: multiple	Shape_lesion_: round/oval	n = 5
Spleen: n = 24 articles; n = 31 descriptions									
1	Echo_lesion_: hypo	n = 24	Echo_lesion_: hypo	Number_lesion_: multiple	n = 19	Size_lesion_: small	Echo_lesion_: hypo	Number_lesion_: multiple	n = 9
2	Number_lesion_: multiple	n = 22	Size_lesion_: small	Echo_lesion_: hypo	n = 10	Location_lesion_: dissem.	Echo_lesion_: hypo	Number_lesion_: multiple	n = 7
3	Size_lesion_: small	n = 11	Size_lesion_: small	Number_lesion_: multiple	n = 10	Echo_lesion_: hypo	Number_lesion_: multiple	Size_organ_: enlarged	n = 6
4	Margin_lesion_: well-defined	n = 11	Location_lesion_: dissem.	Echo_lesion_: hypo	n = 8	Size_lesion_: small	Number_lesion_: multiple	Size_organ_: enlarged	n = 5
5	Location_lesion_: dissem.	n = 9	Location_lesion_: dissem.	Number_lesion_: multiple	n = 8	Size_lesion_: small	Location_lesion_: dissem.	Echo_lesion_: hypo	n = 4
Pericardium: n = 21 articles; n = 26 descriptions									
1	Peri_fluid_: large	n = 15	Peri_fluid_: large	Structure_fluid_: strands	n = 9	Peri_fluid_: large	Echo_fluid_: mixed	Structure_fluid_: strands	n = 4
2	Structure_fluid_: strands	n = 12	Peri_fluid_: large	Echo_fluid_: mixed	n = 8	Peri_fluid_: large	Structure_fluid_: strands	Pericardium: thickening	n = 3
3	Echo_fluid_: mixed	n = 9	Peri_fluid_: large	Pericardium: thickening	n = 5				
4	Peri_fluid_: present	n = 7	Echo_fluid_: mixed	Structure_fluid_: strands	n = 5				
5	Structure_fluid_: mass	n = 7	Peri_fluid_: large	Structure_fluid_: mass	n = 4				
Lung: n = 15 articles; n = 24 descriptions									
1	Echo_cons_: hypo	n = 7	Echo_cons_: hypo	Shape_cons_: round	n = 4	Echo_cons_: hypo	Shape_cons_: round	Size_cons_: <1cm	n = 3
2	Number_cons_: multiple	n = 6	P.line: irregular	Vertical: B-lines	n = 4	Echo_cons_: hypo	Shape_cons_: round	Post. phen: enh.	n = 3
3	Shape_cons_: round	n = 5	P.line: irregular	Location_lesion_: multiple	n = 4	Echo_cons_: hypo	Size_cons_: <1cm	Post. phen: enh.	n = 3
4	Size_cons_: <1cm	n = 5	Echo_cons_: hypo	Size_cons_: <1cm	n = 3	Shape_cons_: round	Size_cons_: <1cm	Post. phen: enh.	n = 3
5	Size_cons_: >1cm	n = 5	Echo_cons_: hypo	Post. phen: enh.	n = 3	P.line: irregular	Vertical: B-lines	Location_lesion_: multiple	n = 3

This table is an abridged version of Supplementary Table 2. We limited organs to those discussed in the Results section and to the 5 most common ultrasound features. All organs and the 10 most common features are available in Supplementary Table 2.

*Number*: number of aspect described (single, multiple: multiple, number not specified); Number_lesion_: number of lesions; Number_cons_: number of lung consolidations.

*Echo*: echogenicity (hypo: hypoechoic, hyper: hyperechoic, mixed: both hyper- and hypoechoic aspects); Echo_lesion_: echogenicity of lesions; Echo_cons_: echogenicity of lung consolidation (mixed: hypo-/hyperechoic aspects, air bronchogram possible); Echo_fluid_: echogenicity of pericardial fluid.

*Size*: Size_organ_: size of organ; Size_cons_: size of consolidation; Size_lesion_: size of lesions (breast: <5 cm small; >5 cm large; spleen, liver: <1 cm small; 1–5 cm medium; >5 cm large).

*Shape*: round: round or oval or nodular; irregular: irregular or no clear shape; lobulated: lobulated or landscape-like; Shape_lesion_: shape of lesions; Shape_cons_: shape of lung consolidations (round: round or oval; shred: no clear shape with shredded or irregular posterior border; mixed: no uniform pattern, variable shapes).

*Others*: Margin_lesion_: margin quality of lesions (for bones: ill-defined includes bone erosions); Location_lesion_: location of lesions (localized: localized or limited to one area; dissem.: disseminated, not limited, multiple areas affected; for prostate and seminal glands: periph.: peripheral prostate areas); Organ_other_: other organs affected (general: continuous: lesion extends to surrounding tissues, eg, muscle, skin; proximity: proximal organs affected eg, neighboring organs; distant: distant organs affected, no direct neighborhood); Peri_fluid_: pericardial fluid; Structure_fluid_: structured content of pericardial fluid (strands: stranding or linear fibrinous elements; mass: mass-like organized lesion); P.line: pleural line pathologies (irregular: irregular line; gap: interrupted pleural line adjacent to consolidation); Vertical: vertical lung ultrasound artefacts; Pericardium: changes in the pericardium itself (thickening: generalized laminar thickening; nodular: focal or nodular thickening); Post. phen.: posterior phenomena (enh.: enhancement).

Table 2 provides example quotations of ultrasound pattern descriptions from included publications as well as our author summary (archetypal ultrasound description) derived subjectively from ranking data (Supplementary Table 2) for each TB-affected organ.

**Table 2.** Author Summary for Typical Findings in Organs (Derived From Extracts in Supplementary Table 2) and Examples From Included Publications

Organ	Example Quotation From Included Papers	Author Summary Derived From Extracted Descriptions
Breast	“a complex mass mainly composed of cystic components, with posterior acoustic enhancement extending to the breast skin and forming a sinus” [9]	“singular lesions in the breast which have heterogenous echotexture and are ill-defined with posterior enhancement, suggestive of abscess formation, often with surrounding infiltration”
Liver	“multiple scattered hypoechoic 5–10-mm lesions in both hepatic lobes […] and the spleen” [10]	“(1) multiple small hypoechoic nodular lesions disseminated across the liver; (2) single, localized lesions with heterogeneous echotexture and ill-defined margins”
Pancreas	“ill-defined hypoechoic 20 ∗ 30 mm mass of the uncinate process with no vascular invasion” [11]	“single large lesions with ill-defined borders in one part of the pancreas with hypoechoic or mixed echogenicity”
Testis	“miliary pattern […] multiple small hypoechoic nodules in diffusely enlarged left testis […] with hydrocele […] and thickening of left scrotal skin” [12]	“multiple hypoechoic round lesions in an often enlarged testis with frequent involvement of neighboring organs”
Epididymis	“Right epididymis was bulky, hypoechoic […], and hypervascular […] nodular enlarged and hypoechoic head and tail region” [13]	“enlarged epididymis with heterogenous echotexture with single large lesion with mixed echogenicity, frequent involvement of neighboring organs like testes”
Spleen	“an enlarged spleen with multiple small hypoechoic round lesions […] These lesions have different sizes but are all less than 1 cm in diameter” [14]	“multiple hypoechoic lesions which are well-defined and disseminated in an often enlarged spleen”
Peripheral lymph nodes	“multiple enlarged nodes […] with heterogeneous echogenicity, intranodal necrosis, nodal matting, and indistinct nodal border” [15]	“(1) multiple nodes with heterogeneous echogenicity and necrosis zones, often matted; (2) single enlarged node with hypoechoic echogenicity and possibly necrosis zones”
Peritoneum or omentum	“diffuse peritoneal thickening and loculated lateral small-volume ascites with thick adhesions” [16]	“hallmark is ascites, frequently stranded, peritoneal generalized thickening mostly involves omentum and often has nodular appearance”
Kidney	“a tuberculous cavity with fine septae within, in the lower part of the left kidney […] Note marked urothelial thickening in this dilated system” [17]	“variable pattern with frequent signs of urostasis or urothelial changes and/or with localized parenchymal lesions which may be singular or multiple with heterogeneous shape and echotexture”
Pericardium	“free floating multiple round discoid echogenic masses in the large amount of pericardial effusion, the thickened pericardium and shaggy echo-dense appearance surrounding the epicardium” [18]	“pericardial effusion (amount large if specified) with mixed echogenicity, often with stranding, occasionally with mass-like structures within; the pericardium is occasionally thickened”
Esophagus	“full-thickness involvement of esophageal wall by a heterogeneous hypoechoic mass, with disruption of the adventitia. […] enlarged heterogeneous hypoechoic mediastinal lymph node infiltrating the esophageal wall, with interior hyperechoic strands” [19]	“esophageal wall architecture disruptions with thickening and mixed-echogenicity lesions which are often contiguous with surrounding lymph nodes”
Intrathoracic lymph nodes	“multiple heteroechoic lymph nodes of varying size (5–20 mm) in the subcarinal space […]. Another predominantly hypoechoic lymph node was found near the ascending aorta. Both the lymph node groups were contiguous with a collection of moving inhomogenous necrotic material” [20]	“multiple enlarged lymph nodes, often with calcification; the esophagus is often simultaneously affected, sometimes with direct contact to the ill-defined nodes “
Abdominal lymph nodes	“multiple round, hypoecogenic, homogeneous lymph nodes, the larger ones with a diameter of 1.5 cm, localized in the hepatic and splenic hilum and peripancreatic region” [21]	“multiple enlarged hypoechoic lymph nodes, often with round shape”
Lung	“Discontinuities of the pleuro-pulmonary surface. Area of parenchyma condensation (hypoechoic) and small amount of fluid between pleura sheets” [22]	“round, hypoechoic consolidations with posterior enhancement or pleural line irregularities with B-line pattern in multiple lung zones”
Intestinal	“small-bowel stricture […] of the left lower quadrant […] a long, circumferential thickening of the jejunum with adjacent involved nodes […] Radial extension of the echogenic luminal contents into the thickened wall […] represent ulcerations” [23]	“thickening of intestinal wall (sometimes long stretch and circumferential) with variable additional findings like perfusion abnormalities, constriction, additional focal lesions or abdominal lymphadenopathy”
Thyroid	“a nodular lesion involving most of the left lobe of thyroid, the left lobe of thyroid was enlarged, measuring 44 mm × 45mm × 33 mm in size. The lesion was hypoechoic and heterogeneous in echogenicity. The hypoechoic areas did not extend into the isthmus. poor blood flow signal was found in the lesion” [24]	“singular lesions >1 cm with hypoechoic or heterogeneous echogenicity with round shape”
Pleura	“pleural thickening adjoining a complex pleural effusion with multiple thin septations” [25]	“a mixed-echogenicity pleural effusion with stranding and sometimes thickening of the pleura”
Prostate and seminal glands	“massive enlargement of prostate with central avascular necrotic area showing moving internal echoes” [26]	“hypoechoic oval singular lesions in the oftentimes enlarged prostate”
Bone	“a heterogeneous collection measuring 4.4 × 3.7 cm surrounded by a thick irregular wall in the right posterolateral chest wall with evidence of few loose bony fragments and underlying bone destruction “ [27]	“single lesions with mixed echogenicity involving the surrounding soft tissues”
Myocardium	“a large mass extending caudally across the tricuspid valve, with a smooth surface and attached to the roof and to the anterolateral wall of the right atrium […] this was characterized by a heterogeneous echogenicity with areas of echolucency” [28]	“single lesions >1 cm which affect myocardium and often endocardial structures like valves, the echogenicity is mostly mixed”
Ovaries	“hypoechoic complex right adnexal mass measuring 70 × 60mm” [29]	“single/localized mixed-echogenicity lesions which are often ill-defined and large in size”
Uterus	“uterus of increased volume with a thickened and non-homogeneous endometrium infiltrating one-third of the myometrium” [30]	“irregular or nodular change of the endometrial lining with thickening and heterogeneous echogenicity, sometimes non-endometrial lesions and also involvement of surrounding organs”
Eyes	“sclerochoroidal thickening with widening of sub-Tenon space with scant fluid and elevation of optic papilla” [31]	“localized pathology in the strata of the eye wall structures with variable additional findings like retinal detachment or choroidal thickening”

Presenting data on all 23 TB-affected organs is beyond the scope of this article. However, for illustration, the female breast (the most commonly described organ) exhibited the following most common sonographic features of TB: “single lesion” (28/45 [62%]) and “mixed echogenicity” (24/45 [53%] of lesions). The most frequent pairs were “single lesion” and “mixed echogenicity” (19/45 [42%]) and “single lesion” and “ill-defined margins” (12/45 [27%]). The most common triad included “mixed echogenicity,” “single lesion,” and “ill-defined margins” (9/45 [20%]).

Data and respective discussion of data relating to the FASH protocol (ie, liver, spleen, abdominal lymph nodes, pleural and pericardial effusions), lung ultrasound, and the esophagus are provided in Supplementary Document 1*E*.

### Sensitivity Analyses

We performed post hoc sensitivity analyses for the ultrasound feature ranking by (1) including only reports with confirmed TB (Supplementary Table 3*A*) and (2) including only reports with confirmed TB and low risk of representation bias (Supplementary Table 3*B*). In summary, stratified data for confirmed TB show subtle changes without significantly altering the rankings for most organs. However, the analysis limited to confirmed TB with low risk for representation bias includes only a few entries.

## DISCUSSION

This systematic review is the first comprehensive attempt to compile sonographic descriptions of TB disease. In some organs (eg, spleen, thyroid), features and their combinations fall into clusters (eg, multiple small lesions in the spleen or single large lesions in the thyroid; see Table 1 and Supplementary Table 2). In other organs, no such clear clusters are revealed (eg, eyes, uterus). The disaggregation of ultrasound features highlights the complexity of medical imaging and carved out specific examples that can be included in future detailed descriptions to enhance comparability (Supplementary Table 1).

In the context of ultrasound findings during TB workup, there remains a significant gap in evidence regarding sonographic patterns indicating TB. TB-related ultrasound descriptions are often derived solely from case reports or case series. However, the example descriptions of organ-associated TB in our synthesis (Supplementary Table 2) demonstrate the potential utility of this data as a clinical or research resource. It is important to note, that data on specificity are rarely included and some publications have shown that TB-like ultrasound findings can also be attributed to non-TB conditions (eg, malignancy or Kikuchi disease causing peripheral lymph nodes [32], or pancreatic lesions caused by adenocarcinoma [33]).

The example of the female breast suggests that mammary TB on ultrasound mostly presents as singular lesions with heterogenous echotexture appearing abscess-like with surrounding infiltration. Another pattern with regular/oval and well-defined lesions is reported with lower frequency. These sonographic patterns warrant clinical awareness in endemic regions.

### Limitations and Strengths

There was high risk of bias from included studies pertaining to selection and ascertainment, stemming from the predominant restriction of ultrasound descriptions to case reports. Sensitivity analyses suggested no relevant changes for confirmed TB but only a few articles were of higher quality, underlining the scarcity of articles in the field (Supplementary Table 3*A* and 3*B*).

Our attempt to translate extracted quotations into respective standardized sonographic features required some interpretation of descriptions, which may in part be subjective. However, our approach was warranted to enable comparison across a wide range of publications.

We analyzed data on a number-of-descriptions basis, not on the number of individuals affected (see Methods). With only a minority of publications reporting prevalence (or reliable accuracy data), this approach avoids overrepresentation of the few features investigated in larger cohorts or studies. While not allowing for epidemiological conclusions, this highlights the clinical focus of the authors' choosing to publish an ultrasound description.

The initial search has not been updated for operational reasons, but it is unlikely that studies published afterward significantly alter the outcome, with current TB ultrasound research focusing on accuracy of known ultrasound features and no relevant changes to ultrasound technology.

The languages included may result in potential underrepresentation of certain regions, particularly LMICs in Africa (see Supplementary Document 3). This review was conducted by clinical point-of-care ultrasound experts; radiologist involvement would be essential for future studies evaluating sonographic patterns systematically.

In conclusion, our review synthesizes available data on ultrasound patterns for TB disease and constitutes an extensive overview of the literature for future prospective and representative studies to assess their relevance in TB diagnostic applications. Our review underlines the need for precise language when describing ultrasound features to increase comparability.

## Supplementary Material

ofaf010_Supplementary_Data

## Supplementary Data

Supplementary materials are available at *Open Forum Infectious Diseases* online. Consisting of data provided by the authors to benefit the reader, the posted materials are not copyedited and are the sole responsibility of the authors, so questions or comments should be addressed to the corresponding author.

## Notes

***Author contributions.*** S. F. W.: Conceptualization, methodology, investigation, writing–original draft. K. M.: Methodology, investigation, writing–original draft. K. K., L. R., and M. G.: Investigation, writing–review and editing. F. T.: Formal analysis, writing–review and editing. S. B.: Conceptualization, writing–review and editing. C. M. D.: Conceptualization, supervision, writing–review and editing.

***Disclaimer.*** The funders had no role in the review design, data collection, data analysis, data interpretation, report writing, or the decision to submit the manuscript for publication. No payment was received by any pharmaceutical company or other agency for the writing of this manuscript.

***Financial support*.** This project was supported by a grant by Deutsches Zentrum für Infektionsforschung (German Center for Infectious Disease Research) (Flexfund ID TTU 02.911). Research reported in this publication was supported by the National Institute of Allergy and Infectious Diseases of the US National Institutes of Health (award number U01AI152087).

## References

[1] World Health Organization . Global tuberculosis report 2023. Geneva, Switzerland: World Health Organization, 2023.

[2] Bélard S, Tamarozzi F, Bustinduy AL, et al Point-of-care ultrasound assessment of tropical infectious diseases—a review of applications and perspectives. Am J Trop Med Hyg 2016; 94:8–21. 26416111 10.4269/ajtmh.15-0421 PMC4710450

[3] Kaminstein D . Point-of-care ultrasound for tropical disease: implications for clinical decision-making. Am J Trop Med Hyg 2020; 103:542–3. 32372745 10.4269/ajtmh.20-0303 PMC7410431

[4] Van Hoving DJ, Griesel R, Meintjes G, Takwoingi Y, Maartens G, Ochodo EA. Abdominal ultrasound for diagnosing abdominal tuberculosis or disseminated tuberculosis with abdominal involvement in HIV-positive individuals. Cochrane Database Syst Rev 2019; 9:CD012777. 31565799 10.1002/14651858.CD012777.pub2 PMC6766789

[5] Bigio J, Kohli M, Klinton JS, et al Diagnostic accuracy of point-of-care ultrasound for pulmonary tuberculosis: a systematic review. PLoS One 2021; 16:e0251236. 33961639 10.1371/journal.pone.0251236 PMC8104425

[6] Bernabeu-Wittel M, Villanueva JL, Pachón J, et al Etiology, clinical features and outcome of splenic microabscesses in HIV-infected patients with prolonged fever. Eur J Clin Microbiol Infect Dis 1999; 18:324–9. 10421038 10.1007/pl00015013

[7] Heller T, Wallrauch C, Goblirsch S, Brunetti E. Focused assessment with sonography for HIV-associated tuberculosis (FASH): a short protocol and a pictorial review. Crit Ultrasound J 2012; 4:21. 23171481 10.1186/2036-7902-4-21 PMC3554543

[8] Murad MH, Sultan S, Haffar S, Bazerbachi F. Methodological quality and synthesis of case series and case reports. BMJ Evid Based Med 2018; 23:60–3. 10.1136/bmjebm-2017-110853 PMC6234235 29420178

[9] Zhang W, Zhang Y, Yang G, Yu T. Features of breast tuberculosis determined by ultrasound imaging: report of 45 cases. J Int Med Res 2020; 48:300060520910891. 32727242 10.1177/0300060520910891 PMC7394031

[10] Grover SB, Taneja DK, Bhatia A, Chellani H. Sonographic diagnosis of congenital tuberculosis: an experience with four cases. Abdom Imaging 2000; 25:622–6. 11029096 10.1007/s002610000071

[11] Jemni I, Akkari I, Mrabet S, Jazia EB. Isolated pancreatic tuberculosis mimicking pancreatic cancer in an immunocompetent host: an elusive diagnosis. Radiol Case Rep 2020; 15:1575–8. 32685072 10.1016/j.radcr.2020.06.041 PMC7355988

[12] Muttarak M, Peh WCG, Lojanapiwat B, Chaiwun B. Tuberculous epididymitis and epididymo-orchitis. AJR Am J Roentgenol 2001; 176:1459–66. 11373214 10.2214/ajr.176.6.1761459

[13] Andrew D, Johny J, Shyam K. Ultrasound findings in a patient with tuberculous epididymo-orchitis. BMJ Case Rep 2020; 13:e237832. 10.1136/bcr-2020-237832 PMC7677334 33208310

[14] Pérez-Solís D, Luyando LH, Callejo-Ortea A, Crespo-Hernández M. Case 90: disseminated tuberculosis. Radiology 2006; 238:366–70. 16373779 10.1148/radiol.2381031758

[15] Moon IS, Kim DW, Baek HJ. Ultrasound-based diagnosis for the cervical lymph nodes in a tuberculosis-endemic area. Laryngoscope 2015; 125:1113–7. 25388942 10.1002/lary.25030

[16] Almajthoub Z, AlNabulsi M, Sharma V. A 58-year-old man with abdominal ascites. Chest 2021; 159:e61–3. 33422244 10.1016/j.chest.2020.01.058

[17] Merchant S, Bharati A, Merchant N. Tuberculosis of the genitourinary system–urinary tract tuberculosis: renal tuberculosis—part I. Indian J Radiol Imaging 2013; 23:46–63. 23986618 10.4103/0971-3026.113615 PMC3737618

[18] Yoon S-A, Hahn Y-S, Hong JM, Lee O-J, Han H-S. Tuberculous pericarditis presenting as multiple free floating masses in pericardial effusion. J Korean Med Sci 2012; 27:325–8. 22379347 10.3346/jkms.2012.27.3.325 PMC3286783

[19] Tang Y, Shi W, Sun X, Xi W. Endoscopic ultrasound in diagnosis of esophageal tuberculosis: 10-year experience at a tertiary care center. Dis Esophagus 2017; 30:1–6. 10.1093/dote/dox031 28575247

[20] Sharma M, Somasundaram A, Mahadevan B. Image of the month. Endoscopic ultrasound in a case of a widened mediastinum. Clin Gastroenterol Hepatol 2010; 8:e94. 20215065 10.1016/j.cgh.2010.02.017

[21] Saieg MA, Yazawa F, Horta M, Rossini LG, Fracassi MT, Del Carlo Bernardi F. The utility of endoscopic ultrasound guided fine needle aspiration in the diagnosis of infectious diseases—report of three cases. Case Rep Infect Dis 2013; 2013:512182. 24392229 10.1155/2013/512182 PMC3872382

[22] Gavrila IL, Badea RI, Jude C, Socaciu MA, Comsa M, Badea AF. Ultrasound as the first imaging method in severe lung disease. Considerations about a case of pulmonary tuberculosis and review of the literature. Med Ultrason 2020; 22:102–4. 32096796 10.11152/mu-1890

[23] Batra A, Gulati MS, Sarma D, Paul SB. Sonographic appearances in abdominal tuberculosis. J Clin Ultrasound 2000; 28:233–45. 10800002 10.1002/(sici)1097-0096(200006)28:5<233::aid-jcu5>3.0.co;2-c

[24] Jiang YD, Gao JQ, Chen S, Deng XY. Thyroid tuberculosis: two cases report and review of the literature. Int J Clin Exp Med 2016; 9:22477–85.

[25] Schlesinger SA, Perera P. Tuberculous pleural effusion. West J Emerg Med 2012; 13:313–4. 22942929 10.5811/westjem.2011.9.6846 PMC3421969

[26] Baral S, Chhetri RK, Gyawali M, et al Prostate tuberculosis complicated by huge prostatic abscess: a rare case report from Nepal. Int J Surg Case Rep 2020; 77:152–6. 33161288 10.1016/j.ijscr.2020.10.045 PMC7649586

[27] Sharma BK, Singh VK, Nishant K, Das D. Scapular bone destruction: do not forget to think of tuberculosis in endemic areas. BMJ Case Rep 2013; 2013:bcr2013200051. 10.1136/bcr-2013-200051 PMC3736681 23833009

[28] Mondoni M, Centola M, Viganò O, et al Chest pain and a left parasternal soft tissue swelling in an immunocompetent refugee with disseminated tuberculosis. Int J Infect Dis 2020; 90:116–8. 31693940 10.1016/j.ijid.2019.10.033

[29] Akhtar N, Hayat Z, Nazim F. Genital tuberculosis mimicking carcinoma ovary: can ultrasound guided biopsy be a resolution? J Ayub Med Coll Abbottabad 2017; 29:496–8. 29076692

[30] Iovenitti P, Ruggeri G, Tatangelo R, Palermo P, Carta G. Endometrial tuberculosis: a clinical case. Clin Exp Obstet Gynecol 2011; 38:186–7. 21793289

[31] Agarwal AM, Dutta Majumder P. Tubercular posterior scleritis: a case report and review of literature. Indian J Ophthalmol 2019; 67:1362–5. 31332144 10.4103/ijo.IJO_1942_18 PMC6677048

[32] Park JH, Kim DW. Sonographic diagnosis of tuberculous lymphadenitis in the neck. J Ultrasound Med 2014; 33:1619–26. 25154944 10.7863/ultra.33.9.1619

[33] Rana SS, Bhasin DK, Srinivasan R, Sampath S, Mittal BR, Singh K. Distinctive endoscopic ultrasound features of isolated pancreatic tuberculosis and requirements for biliary stenting. Clin Gastroenterol Hepatol 2012; 10:323–5. 22037426 10.1016/j.cgh.2011.10.018

