# Supplementary material for: Tuberculosis Visualized With the Ultrasound Probe: A Systematic Review of Sonographic Pattern Descriptions and an Analysis of Common Sonographic Features
Source: Open Forum Infect Dis. 2025 Mar 7;12(3):ofaf010. doi: 10.1093/ofid/ofaf010 (PMC11886843; doi:10.1093/ofid/ofaf010)
Supplement: ofaf010_Supplementary_Data [file ofaf010_supplementary_data.zip › 2 S1 SR TB Supplement mixed_16122024_clean.docx]

**Supplemental Document 1 – supplemental materials**

**Title:** Tuberculosis visualized with the ultrasound probe – a systematic review of sonographic pattern descriptions and an analysis of common sonographic features

**Authors:** Stefan Fabian Weber^1,2,3^*, Katharina Manten^4,3^*, Katharina Kleiber^5^, Lisa Ruby^6,7^, Maurizio Grilli^8^, Frank Tobian^1,3^, Sabine Bélard^6,7*^, Claudia M. Denkinger^1,3#*^

1 University Hospital Heidelberg, Department for Infectious Disease and Tropical Medicine

2 University Hospital Heidelberg, Department for Parasitology

3 German Center for Infectious Disease Research, DZIF Partner Site Heidelberg

4 University Hospital Heidelberg, Department of Anaesthesiology

5 Evangelisches Krankenhaus Köln-Kalk, Internal Medicine

6 University of Tübingen, Institute of Tropical Medicine

7 German Center for Infectious Disease Research, DZIF Partner Site Tübingen

8 Library of the Medical Faculty Mannheim of the University of Heidelberg, University Medical Center Mannheim

* contributed equally

^#^ corresponding author: Claudia M. Denkinger (Department for Infectious Diseases and Tropical Medicine, University Hospital Heidelberg, Im Neuenheimer Feld 324, 69120 Heidelberg, Tel.: +49 6221 56 22999, [Claudia.denkinger@uni-heidelberg.de](mailto:Claudia.denkinger@uni-heidelberg.de))

**Supplemental Document 1 – supplemental materials (this document)**

1. Supplement Table S1 (attributes extracted)
2. Supplement Table S2 expanded, single ultrasound features, pairs and triads by organ and frequency
3. Supplement Table S3a (sensitivity analysis only confirmed TB)
4. Supplement Table S3b (sensitivity analysis only confirmed TB and representative)
5. Exemplary results and discussion of selected organ descriptions

Other supplemental materials (other documents):

Document S2: Search protocol

Document S3: Excel with all papers incl. QUADAS, ultrasound patterns and feature extraction

Document S4: PRISMA Checklist incl. abstract checklist

Document S5: Examples for papers which appear to meet inclusion criteria but which were excluded

**a) Supplement Table 1 features extracted per organ**

| General aspects | Pertaining to the organ parenchyma itself   - General echogenicity and changes from normal - Changes in size of organ (e.g., enlargement) - Vascularity - Additional features like calcification   Lesions within an organ   - Size - Echogenicity - Number - Shape - Margin - Posterior phenomena (e.g., shadowing) - Additional features like vascularity, calcification - Location in organ - Echogenicity surrounding the organ   Whether other organs, especially neighboring organs are affected |
| --- | --- |
| Breast | lymphatic vessel abnormalities, breast duct abnormalities, laterality |
| Liver | bile system pathologies |
| Pancreas | bile system pathology, margin of pancreas |
| Testis and epididymis | echogenicity of surrounding tissue, i.e., hydrocele  neighboring organs, e.g., scrotal fistula with skin involvement |
| Lymph nodes  (peripheral, thoracic, abdominal) | Size of largest lymph node, intranodal lesions, number of lymph nodes, shape of lymph nodes, margin of lymph nodes, nodal vascularity pattern, other organs affected (e.g., continuous esophageal lesion in mediastinal lymph nodes), local effect on neighboring organs, presence of ascites or presence of mesentery pathology (abdominal lymph nodes), elastography pattern |
| Peritoneum or omentum | Presence of ascites, amount of ascites, echogenicity of ascites, peritoneal changes, echogenicity of thickened peritoneum, size of peritoneal nodules, echogenicity of peritoneal nodules, echogenicity of peritoneal nodules, location of peritoneal changes, matting of bowels, additional lymphadenopathy, presence of abdominal mass, echogenicity of mass, size of mass, shape of mass |
| Kidney | signs of urostasis, urothelial changes, capsule changes, |
| Pericardium | Presence and size of effusion, echogenicity of effusion, organized content in pericardial space, pericardial changes, echogenicity of organized intrapericardial masses |
| Esophagus | Lesion location in esophageal circumference, echogenicity of esophageal wall, thickness of esophageal wall, extent of esophageal wall affected, esophageal wall architecture, other organs affected (e.g., mediastinal lymph nodes) |
| Lung | Echogenicity of consolidations, shape of consolidation, size of consolidation, number of consolidations, pleural line changes adjacent to consolidation, posterior ultrasound phenomena, general pleural line irregularities, general vertical artifacts, location of pathology, changes in lung movement, pleural space changes |
| Intestinal | Intestinal wall changes, location in intestinal wall circumference, length of affected intestine, echogenicity of affected intestine, intestinal wall architecture, functional changes, matting of bowels, additional lymphadenopathy |
| Pleura | Presence of effusion, size of effusion, echogenicity of effusion, organized content in pleural space, pleural changes, echogenicity of thickened pleura, location of fluid, lung changes |
| Prostate and seminal glands | prostate and/or seminal gland affected |
| Myocardium | cardiomyopathy, pericardial space changes |
| Uterus | Endometrial thickness, endometrial echogenicity, endometrial lining changes |
| Eyes | Changes in the choroideum, sub-tenon changes, papillary changes, level of internal reflectivity |

**b) Supplement Table 2 expanded, single ultrasound features, pairs and triads by organ and frequency**

| **Breast;** No. Papers: n= 25; No. Descriptions: n=45 | | | | | | | | | |
| --- | --- | --- | --- | --- | --- | --- | --- | --- | --- |
|  | ***Single feature*** | | ***Two-feature combinations*** | | | ***Three-feature combinations*** | | | |
| 1 | Number_lesion_: single | n=28 | Number_lesion_: single | Echo_lesion_: mixed | n=19 | Echo_lesion_: mixed | Number_lesion_: single | Margin_lesion_: ill-defined | n=9 |
| 2 | Echo_lesion_: mixed | n=24 | Number_lesion_: single | Margin_lesion_: ill-defined | n=12 | Echo_lesion_: mixed | Number_lesion_: single | Post. phen: enh. | n=7 |
| 3 | Margin_lesion_: ill-defined | n=16 | Margin_lesion_: ill-defined | Echo_lesion_: mixed | n=10 | Echo_lesion_: mixed | Margin_lesion_: ill-defined | Post. phen: enh. | n=6 |
| 4 | Echo_lesion_: hypo | n=15 | Echo_lesion_: hypo | Number_lesion_: single | n=9 | Echo_lesion_: mixed | Number_lesion_: single | Margin_lesion_: well-defined | n=5 |
| 5 | Shape_lesion_: round/oval | n=12 | Echo_lesion_: mixed | Post. phen: enh. | n=9 | Echo_lesion_: mixed | Number_lesion_: single | Organ_other_: continuous | n=5 |
| 6 | Post. phen: enh. | n=11 | Number_lesion_: single | Shape_lesion_: round/oval | n=8 | Number_lesion_: single | Margin_lesion_: ill-defined | Post. phen: enh. | n=5 |
| 7 | Organ_other_: continuous | n=11 | Number_lesion_: single | Post. phen: enh. | n=8 | Number_lesion_: single | Size_lesion_: large | Echo_lesion_: mixed | n=4 |
| 8 | Location_lesion_: localized | n=10 | Location_lesion_: localized | Number_lesion_: single | n=7 | Location_lesion_: localized | Echo_lesion_: mixed | Number_lesion_: single | n=4 |
| 9 | Margin_lesion_: well-defined | n=10 | Number_lesion_: single | Margin_lesion_: well-defined | n=7 | Shape_lesion_: round/oval | Number_lesion_: single | Echo_lesion_: hypo | n=4 |
| 10 | Number_lesion_: multiple | n=7 |  | | | Echo_lesion_: mixed | Number_lesion_: single | Shape_lesion_: round/oval | n=4 |
| **Liver;** No. Papers: n= 30; No. descriptions: n=40 | | | | | | | | | |
| 1 | Echo_lesion_: hypo | n=24 | Echo_lesion_: hypo | Number_lesion_: multiple | n=14 | Size_lesion_: small | Echo_lesion_: hypo | Number_lesion_: multiple | n=8 |
| 2 | Number_lesion_: multiple | n=21 | Echo_lesion_: hypo | Shape_lesion_: round/oval | n=11 | Location_lesion_: dissem. | Echo_lesion_: hypo | Number_lesion_: multiple | n=8 |
| 3 | Shape_lesion_: round/oval | n=17 | Location_lesion_: dissem. | Number_lesion_: multiple | n=10 | Size_lesion_: small | Location_lesion_: dissem. | Echo_lesion_: hypo | n=5 |
| 4 | Location_lesion_: localized | n=15 | Location_lesion_: localized | Number_lesion_: single | n=9 | Size_lesion_: small | Location_lesion_: dissem. | Number_lesion_: multiple | n=5 |
| 5 | Number_lesion_: single | n=12 | Number_lesion_: multiple | Shape_lesion_: round/oval | n=9 | Location_lesion_: dissem. | Number_lesion_: multiple | Shape_lesion_: round/oval | n=5 |
| 6 | Echo_lesion_: mixed | n=11 | Size_lesion_: small | Echo_lesion_: hypo | n=8 | Echo_lesion_: hypo | Number_lesion_: multiple | Shape_lesion_: round/oval | n=5 |
| 7 | Margin_lesion_: ill-defined | n=11 | Size_lesion_: small | Number_lesion_: multiple | n=8 |  | | | |
| 8 | Location_lesion_: dissem. | n=10 | Location_lesion_: dissem. | Echo_lesion_: hypo | n=8 |  |  |  |  |
| 9 | Size_lesion_: small | n=8 | Location_lesion_: localized | Echo_lesion_: hypo | n=7 |  |  |  |  |
| 10 | Size_lesion_: medium | n=7 | Location_lesion_: localized | Margin_lesion_: ill-defined | n=7 |  |  |  |  |
| **Pancreas;** No. Papers: n= 34; No. Descritpions: n= 37 | | | | | | | | | |
| 1 | Number_lesion_: single | n=30 | Number_lesion_: single | Size_lesion_: large | n=21 | Number_lesion_: single | Size_lesion_: large | Location_lesion_: localized | n=14 |
| 2 | Size_lesion_: large | n=21 | Number_lesion_: single | Location_lesion_: localized | n=19 | Number_lesion_: single | Size_lesion_: large | Echo_lesion_: mixed | n=11 |
| 3 | Echo_lesion_: hypo | n=20 | Echo_lesion_: hypo | Number_lesion_: single | n=16 | Location_lesion_: localized | Echo_lesion_: hypo | Number_lesion_: single | n=11 |
| 4 | Location_lesion_: localized | n=19 | Location_lesion_: localized | Size_lesion_: large | n=14 | Size_lesion_: large | Echo_lesion_: hypo | Number_lesion_: single | n=10 |
| 5 | Echo_lesion_: mixed | n=14 | Echo_lesion_: mixed | Number_lesion_: single | n=14 | Size_lesion_: large | Location_lesion_: localized | Echo_lesion_: hypo | n=8 |
| 6 | Margin_lesion_: ill-defined | n=12 | Size_lesion_: large | Echo_lesion_: mixed | n=11 | Number_lesion_: single | Size_lesion_: large | Margin_lesion_: ill-defined | n=8 |
| 7 | Margin_lesion_: well-defined | n=10 | Location_lesion_: localized | Echo_lesion_: hypo | n=11 | Location_lesion_: localized | Echo_lesion_: mixed | Number_lesion_: single | n=8 |
| 8 | Location_lesion_: dissem. | n=7 | Number_lesion_: single | Margin_lesion_: ill-defined | n=11 | Location_lesion_: localized | Number_lesion_: single | Margin_lesion_: ill-defined | n=7 |
| 9 | Shape_lesion_: lobulated | n=6 | Size_lesion_: large | Echo_lesion_: hypo | n=10 | Size_lesion_: large | Location_lesion_: localized | Echo_lesion_: mixed | n=6 |
| 10 | Vasc.: decreased | n=6 |  | | | | | | |
| **Testis:** No. Papers: n= 21; No. Descriptions: n=36 | | | | | | | | | |
| 1 | Echo_lesion_: hypo | n=21 | Echo_lesion_: hypo | Number_lesion_: multiple | n=17 | Echo_lesion_: hypo | Number_lesion_: multiple | Shape_lesion_: round/oval | n=10 |
| 2 | Number_lesion_: multiple | n=21 | Echo_lesion_: hypo | Organ_other_: proximity | n=12 | Echo_lesion_: hypo | Number_lesion_: multiple | Organ_other_: proximity | n=10 |
| 3 | Organ_other_: proximity | n=19 | Number_lesion_: multiple | Shape_lesion_: round/oval | n=12 | Echo_lesion_: hypo | Number_lesion_: multiple | Size_organ_: enlarged | n=9 |
| 4 | Size_organ_: enlarged | n=17 | Echo_lesion_: hypo | Shape_lesion_: round/oval | n=11 | Number_lesion_: multiple | Shape_lesion_: round/oval | Size_organ_: enlarged | n=7 |
| 5 | Shape_lesion_: round/oval | n=15 | Number_lesion_: multiple | Size_organ_: enlarged | n=11 | Number_lesion_: multiple | Shape_lesion_: round/oval | Organ_other_: proximity | n=7 |
| 6 | Surround.: fluid | n=12 | Number_lesion_: multiple | Organ_other_: proximity | n=11 | ** | | | |
| 7 | Number_lesion_: single | n=12 | Echo_lesion_: hypo | Size_organ_: enlarged | n=10 |  |  |  |  |
| 8 | Echo_lesion_: mixed | n=11 | Size_organ_: enlarged | Organ_other_: proximity | n=9 |  |  |  |  |
| 9 | Vasc.: increased | n=10 | * | | |  |  |  |  |
| 10 | Echo_organ_: mixed | n=9 |  |  |  |  |  |  |  |
| **Epididymis:** No. papers: n= 22; No. Descriptions: n=35 | | | | | | | | | |
| 1 | Size_organ_: enlarged | n=23 | Size_organ_: enlarged | Echo_organ_: mixed | n=13 | Location_lesion_: localized | Echo_organ_: mixed | Size_organ_: enlarged | n=8 |
| 2 | Location_lesion_: localized | n=20 | Organ_other_: proximity | Size_organ_: enlarged | n=13 | Location_lesion_: localized | Echo_lesion_: mixed | Number_lesion_: single | n=6 |
| 3 | Organ_other_: proximity | n=17 | Size_organ_: enlarged | Location_lesion_: localized | n=12 | Echo_organ_: mixed | Size_organ_: enlarged | Organ_other_: proximity | n=6 |
| 4 | Echo_organ_: mixed | n=14 | Location_lesion_: localized | Number_lesion_: single | n=10 | Location_lesion_: localized | Size_organ_: enlarged | Organ_other_: proximity | n=5 |
| 5 | Number_lesion_: single | n=10 | Location_lesion_: localized | Echo_organ_: mixed | n=8 | Size_organ_: enlarged | Vasc.: increased | Organ_other_: proximity | n=5 |
| 6 | Vasc.: increased | n=9 | Location_lesion_: localized | Organ_other_: proximity | n=8 | Size_lesion_: large | Location_lesion_: localized | Echo_lesion_: mixed | n=4 |
| 7 | Echo_lesion_: mixed | n=7 | Vasc.: increased | Organ_other_: proximity | n=7 | Size_lesion_: large | Location_lesion_: localized | Number_lesion_: single | n=4 |
| 8 | Surround.: fluid | n=5 |  | | | Size_lesion_: large | Echo_lesion_: mixed | Number_lesion_: single | n=4 |
| 9 |  | |  |  |  | Location_lesion_: localized | Number_lesion_: single | Organ_other_: proximity | n=4 |
| 10 |  |  |  |  |  |  | | | |
| **Spleen:** No. Papers: n= 24; No. Descriptions: n=31 | | | | | | | | | |
| 1 | Echo_lesion_: hypo | n=24 | Echo_lesion_: hypo | Number_lesion_: multiple | n=19 | Size_lesion_: small | Echo_lesion_: hypo | Number_lesion_: multiple | n=9 |
| 2 | Number_lesion_: multiple | n=22 | Size_lesion_: small | Echo_lesion_: hypo | n=10 | Location_lesion_: dissem. | Echo_lesion_: hypo | Number_lesion_: multiple | n=7 |
| 3 | Size_lesion_: small | n=11 | Size_lesion_: small | Number_lesion_: multiple | n=10 | Echo_lesion_: hypo | Number_lesion_: multiple | Size_organ_: enlarged | n=6 |
| 4 | Margin_lesion_: well-defined | n=11 | Location_lesion_: dissem. | Echo_lesion_: hypo | n=8 | Size_lesion_: small | Number_lesion_: multiple | Size_organ_: enlarged | n=5 |
| 5 | Location_lesion_: dissem. | n=9 | Location_lesion_: dissem. | Number_lesion_: multiple | n=8 | Size_lesion_: small | Location_lesion_: dissem. | Echo_lesion_: hypo | n=4 |
| 6 | Number_lesion_: single | n=7 | Echo_lesion_: hypo | Margin_lesion_: well-defined | n=7 | Size_lesion_: small | Location_lesion_: dissem. | Number_lesion_: multiple | n=4 |
| 7 | Shape_lesion_: round/oval | n=7 | Number_lesion_: multiple | Size_organ_: enlarged | n=7 | Size_lesion_: small | Echo_lesion_: hypo | Size_organ_: enlarged | n=4 |
| 8 | Size_organ_: enlarged | n=7 | Echo_lesion_: hypo | Size_organ_: enlarged | n=6 | Size_lesion_: small | Number_lesion_: multiple | Shape_lesion_: round/oval | n=4 |
| 9 | Size_lesion_: large | n=6 | Number_lesion_: single | Margin_lesion_: well-defined | n=6 | Echo_lesion_: hypo | Number_lesion_: multiple | Margin_lesion_: ill-defined | n=4 |
| 10 | Location_lesion_: localized | n=5 | Size_lesion_: small | Size_organ_: enlarged | n=5 | Echo_lesion_: hypo | Number_lesion_: multiple | Organ_other_: distant | n=4 |
| **Peripheral lymph nodes;** No. Papers: n= 24 No. Descriptions: n=36 | | | | | | | | | |
| 1 | Vasc.: present | n=16 | Echo_ln_: mixed | Number_ln_: multiple | n=8 | Echo_ln_: mixed | Intranodal: necro | Number_ln_: multiple | n=5 |
| 2 | Echo_ln_: mixed | n=13 | Echo_ln_: mixed | Intranodal: necro | n=7 | Echo_ln_: mixed | Number_ln_: multiple | Margin_ln_: ill-defined | n=5 |
| 3 | Number_ln_: single | n=13 | Size_organ_: enlarged | Number_ln_: single | n=6 | Echo_ln_: mixed | Intranodal: necro | Margin_ln_: ill-defined | n=4 |
| 4 | Intranodal: necro | n=11 | Number_ln_: multiple | Margin_ln_: ill-defined | n=6 | Intranodal: necro | Number_ln_: multiple | Margin_ln_: ill-defined | n=4 |
| 5 | Number_ln_: multiple | n=9 | Echo_ln_: mixed | Margin_ln_: ill-defined | n=5 | Echo_ln_: mixed | Number_ln_: multiple | Shape_ln_: rounded | n=3 |
| 6 | Size_organ_: enlarged | n=8 | Number_ln_: multiple | Intranodal: necro | n=5 | Echo_ln_: mixed | Vasc.: preseved | Vasc.: mixed | n=3 |
| 7 | Shape_ln_: rounded | n=6 |  | | |  | | | |
| 8 | Margin_ln_: ill-defined | n=6 |  |  |  |  |  |  |  |
| 9 |  | |  |  |  |  |  |  |  |
| **Peritoneum and omentum;** No. Papers: n= 19 No. Descriptions: n=35 | | | | | | | | | |
| 1 | Ascites: present | n=17 | Ascites: present | Echo_ascites_: stranding | n=9 | Ascites: present | Amount_ascites_: small | Echo_ascites_: stranding | n=5 |
| 2 | Location_PChanges_: Omentum | n=13 | Peritoneum: thickening | Location_PChanges_: Omentum | n=8 | Peritoneum: thickening | Echo_Peritoneum_: hyper | Location_PChanges_: Omentum | n=4 |
| 3 | Peritoneum: thickening | n=11 | Ascites: present | Amount_ascites_: small | n=6 | Peritoneum: Nodular | Location_PChanges_: Omentum | Echo_PNodules_: Hypo | n=3 |
| 4 | Peritoneum: Nodular | n=10 | Amount_ascites_: small | Echo_ascites_: stranding | n=5 |  | | | |
| 5 | Echo_ascites_: stranding | n=9 | Peritoneum: thickening | Echo_Peritoneum_: hyper | n=4 |  |  |  |  |
| 6 | Amount_ascites_: small | n=6 | Peritoneum: Nodular | Location_PChanges_: Omentum | n=4 |  |  |  |  |
| 7 | Echo_PNodules_: Hypo | n=5 | Peritoneum: Nodular | Echo_PNodules_: Hypo | n=4 |  |  |  |  |
| 8 | Echo_Peritoneum_: hyper | n=4 | Echo_Peritoneum_: hyper | Location_PChanges_: Omentum | n=4 |  |  |  |  |
| 9 |  | | Location_PChanges_: Omentum | Echo_PNodules_: Hypo | n=4 |  |  |  |  |
| **Kidney:** No. Papers: n= 10; No. Descriptions: n=28 | | | | | | | | | |
| 1 | U.tract: urostasis | n=13 | U.tract: urostasis | Urothelium: thickening | n=7 | Echo_lesion_: mixed | Number_lesion_: multiple | Shape_lesion_: irregular | n=3 |
| 2 | Number_lesion_: multiple | n=10 | Location_lesion_: localized | Number_lesion_: single | n=5 | Number_lesion_: multiple | Shape_lesion_: irregular | Margin_Capsule_: ill-defined | n=3 |
| 3 | Location_lesion_: localized | n=9 | Number_lesion_: multiple | Shape_lesion_: irregular | n=4 |  | | | |
| 4 | Urothelium: thickening | n=8 | Number_lesion_: multiple | Margin_Capsule_: ill-defined | n= 4 |  |  |  |  |
| 5 | Number_lesion_: single | n=7 | U.tract: urostasis | Margin_Capsule_: ill-defined | n=4 |  |  |  |  |
| 6 | Margin_Capsule_: ill-defined | n=7 |  | | |  |  |  |  |
| 7 | Calcification: present | n=6 |  |  |  |  |  |  |  |
| 8 | Shape_lesion_: irregular | n=5 |  |  |  |  |  |  |  |
| 9 | Echo_organ_: strata-loss | n=5 |  |  |  |  |  |  |  |
| 10 | Echo_lesion_: mixed | n=5 |  |  |  |  |  |  |  |
| **Pericardium:** No. Papers: n= 21; No. Descriptions: n=26 | | | | | | | | | |
| 1 | Peri_fluid_: large | n=15 | Peri_fluid_: large | Structure_fluid_: strands | n=9 | Peri_fluid_: large | Echo_fluid_: mixed | Structure_fluid_: strands | n=4 |
| 2 | Structure_fluid_: strands | n=12 | Peri_fluid_: large | Echo_fluid_: mixed | n=8 | Peri_fluid_: large | Structure_fluid_: strands | Pericardium: thickening | n=3 |
| 3 | Echo_fluid_: mixed | n=9 | Peri_fluid_: large | Pericardium: thickening | n=5 |  |  |  |  |
| 4 | Peri_fluid_: present | n=7 | Echo_fluid_: mixed | Structure_fluid_: strands | n=5 |  |  |  |  |
| 5 | Structure_fluid_: mass | n=7 | Peri_fluid_: large | Structure_fluid_: mass | n=4 |  |  |  |  |
| 6 | Pericardium: thickening | n=7 | Structure_fluid_: mass | Echo_perimass_: hyper | n=4 |  |  |  |  |
| 7 | Pericardium: nodular | n=4 | Structure_fluid_: strands | Pericardium: thickening | n=3 |  |  |  |  |
| 8 | Echo_perimass_: hyper | n=4 |  |  |  |  |  |  |  |
| **Esophagus**: No. Papers: n=15; No. Descriptions: n=26 | | | | | | | | | |
| 1 | Wall_archi_: disrupted | n=16 | Echo_lesion_: mixed | Number_lesion_: multiple | n=7 | Echo_lesion_: mixed | Number_lesion_: multiple | Margin_lesion_: well-defined | n=6 |
| 2 | Echo_lesion_: mixed | n=13 | Wall_thick_: thickened | Wall_archi_: disrupted | n=7 | Echo_lesion_: mixed | Number_lesion_: single | Wall_archi_: disrupted | n=5 |
| 3 | Med.ln.: continuous | n=12 | Echo_lesion_: mixed | Wall_archi_: disrupted | n=6 |  | | | |
| 4 | Wall_thick_: thickened | n=10 | Echo_lesion_: mixed | Number_lesion_: single | n=6 |  |  |  |  |
| 5 | Number_lesion_: multiple | n=9 | Echo_lesion_: mixed | Margin_lesion_: well-defined | n=6 |  |  |  |  |
| 6 | Number_lesion_: single | n=7 | Wall_archi_: disrupted | Number_lesion_: single | n=6 |  |  |  |  |
| 7 | Margin_lesion_: well-defined | n=6 | Wall_archi_: disrupted | Med.ln.: continuous | n=6 |  |  |  |  |
| 8 | Calcification: present | n=5 | Number_lesion_: multiple | Margin_lesion_: well-defined | n=6 |  |  |  |  |
| 9 | Med.ln.: present | n=5 |  | | |  |  |  |  |
| **Intrathoracic lymph nodes**: No. Papers: n=20; No. Descriptions: n=26 | | | | | | | | | |
| 1 | Number_ln_: multiple | n=15 | Number_ln_: multiple | Margin_ln_: ill-defined | n=9 | Number_ln_: multiple | Margin_ln_: ill-defined | Organ_other_: esophagus | n=6 |
| 2 | Organ_other_: esophagus | n=15 | Number_ln_: multiple | Organ_other_: esophagus | n=9 | Margin_ln_: ill-defined | Intranodal: hyper | Organ_other_: esophagus | n=6 |
| 3 | Echo_ln_: mixed | n=14 | Size_organ_: enlarged | Number_ln_: multiple | n=8 | Size_organ_: enlarged | Echo_ln_: mixed | Number_ln_: multiple | n=5 |
| 4 | Margin_ln_: ill-defined | n=12 | Margin_ln_: ill-defined | Organ_other_: esophagus | n=8 | Size_organ_: enlarged | Number_ln_: multiple | Margin_ln_: ill-defined | n=5 |
| 5 | Size_organ_: enlarged | n=10 | Echo_ln_: mixed | Number_ln_: multiple | n=7 | Size_organ_: enlarged | Number_ln_: multiple | Organ_other_: esophagus | n=5 |
| 6 | Intranodal: hyper | n=10 | Echo_ln_: mixed | Intranodal: hyper | n=7 | Echo_ln_: mixed | Number_ln_: multiple | Intranodal: hyper | n=5 |
| 7 | Surround.: matting | n=8 | Echo_ln_: mixed | Organ_other_: esophagus | n=7 | Number_ln_: multiple | Margin_ln_: ill-defined | Intranodal: hyper | n=5 |
| 8 | Number_ln_: single | n=6 | Number_ln_: multiple | Intranodal: hyper | n=7 | Number_ln_: multiple | Intranodal: hyper | Organ_other_: esophagus | n=5 |
| 9 | Shape_ln_: oval | n=4 | Intranodal: hyper | Organ_other_: esophagus | n=7 |  | | | |
| 10 | Intranodal: necro | n=4 |  | | |  |  |  |  |
| **Abdominal lymph nodes**: No. Papers: n=19; No. Descriptions: n=23 | | | | | | | | | |
| 1 | Size_organ_: enlarged | n=17 | Size_organ_: enlarged | Echo_ln_: hypo | n=10 | Size_organ_: enlarged | Echo_ln_: hypo | Number_ln_: multiple | n=6 |
| 2 | Number_ln_: multiple | n=13 | Size_organ_: enlarged | Number_ln_: multiple | n=10 | Size_organ_: enlarged | Echo_ln_: hypo | Shape_ln_: rounded | n=6 |
| 3 | Echo_ln_: hypo | n=11 | Size_organ_: enlarged | Shape_ln_: rounded | n=7 | Size_organ_: enlarged | Number_ln_: multiple | Shape_ln_: rounded | n=6 |
| 4 | Shape_ln_: rounded | n=7 | Echo_ln_: hypo | Number_ln_: multiple | n=6 | Echo_ln_: hypo | Number_ln_: multiple | Shape_ln_: rounded | n=5 |
| 5 | Location_ln_: mesent. | n=5 | Echo_ln_: hypo | Shape_ln_: rounded | n=6 | Location_ln_: liver | Size_organ_: enlarged | Echo_ln_: hypo | n=3 |
| 6 | Location_ln_: panc. | n=5 | Number_ln_: multiple | Shape_ln_: rounded | n=6 | Location_ln_: mesent. | Size_organ_: enlarged | Number_ln_: multiple | n=3 |
| 7 | Number_ln_: single | n=5 | Size_organ_: enlarged | Number_ln_: single | n=5 | Size_organ_: enlarged | Echo_ln_: hypo | Number_ln_: single | n=3 |
| 8 | Margin_ln_: ill-defined | n=5 | Location_ln_: mesent. | Size_organ_: enlarged | n=4 | Size_organ_: enlarged | Echo_ln_: hypo | Intranodal: necro | n=3 |
| 9 | Intranodal: necro | n=5 | Echo_ln_: hypo | Intranodal: necro | n=4 | Size_organ_: enlarged | Number_ln_: single | Margin_ln_: well-defined | n=3 |
| **Lung**: No. Papers: n=15 No. Descriptions: n=24 | | | | | | | | | |
| 1 | Echo_cons_: hypo | n=7 | Echo_cons_: hypo | Shape_cons_: round | n=4 | Echo_cons_: hypo | Shape_cons_: round | Size_cons_: <1cm | n=3 |
| 2 | Number_cons_: multiple | n=6 | P.line: irregular | Vertical: B-lines | n=4 | Echo_cons_: hypo | Shape_cons_: round | Post. phen: enh. | n=3 |
| 3 | Shape_cons_: round | n=5 | P.line: irregular | Location_lesion_: multiple | n=4 | Echo_cons_: hypo | Size_cons_: <1cm | Post. phen: enh. | n=3 |
| 4 | Size_cons_: <1cm | n=5 | Echo_cons_: hypo | Size_cons_: <1cm | n=3 | Shape_cons_: round | Size_cons_: <1cm | Post. phen: enh. | n=3 |
| 5 | Size_cons_: >1cm | n=5 | Echo_cons_: hypo | Post. phen: enh. | n=3 | P.line: irregular | Vertical: B-lines | Location_lesion_: multiple | n=3 |
| 6 | Post. phen: enh. | n=5 | Shape_cons_: round | Size_cons_: <1cm | n=3 |  | | | |
| 7 | P.line: irregular | n=5 | Shape_cons_: round | Post. phen: enh. | n=3 |  |  |  |  |
| 8 | Vertical: B-lines | n=5 | Shape_cons_: round | Margin_lesion_: well-defined | n=3 |  |  |  |  |
| 9 | Location_lesion_: multiple | n=5 | Size_cons_: <1cm | Post. phen: enh. | n=3 |  |  |  |  |
| 10 |  | | Vertical: B-lines | Location_lesion_: multiple | n=3 |  |  |  |  |
| **Intestines**: No. Papers: n=14; No. Descriptions: n=21 | | | | | | | | | |
| 1 | Wall_thick_: thickening | n=16 | Wall_thick_: thickening | Wall_location_: circum | n=5 |  | | | |
| 2 | Wall_location_: circum | n=5 | Wall_thick_: thickening | Vasc.: mixed | n=5 |  |  |  |  |
| 3 | Vasc.: mixed | n=5 | Wall_thick_: thickening | Passage: constricted | n=4 |  |  |  |  |
| 4 | Passage: constricted | n=4 | Wall_thick_: thickening | Length: long | n=4 |  |  |  |  |
| 5 | Length: long | n=4 | Wall_thick_: thickening | Organ_other_: abd.ln | n=4 |  |  |  |  |
| 6 | Wall_archi_: disrupted | n=4 | Wall_thick_: thickening | Wall_echo_: mixed | n=3 |  |  |  |  |
| 7 | Echo_lesion_: hypo | n=4 | Wall_thick_: thickening | Number_lesion_: multiple | N=3 |  |  |  |  |
| 8 | Organ_other_: abd.ln | n=4 |  | | |  |  |  |  |
| **Thyroid**: No. Papers: n=15; No. Descriptions: n=19 | | | | | | | | | |
| 1 | Number_lesion_: single | n=13 | Size_lesion_: large | Echo_lesion_: mixed | n=9 | Size_lesion_: large | Echo_lesion_: mixed | Number_lesion_: single | n=6 |
| 2 | Size_lesion_: large | n=12 | Size_lesion_: large | Number_lesion_: single | n=9 | Size_lesion_: large | Echo_lesion_: mixed | Shape_lesion_: round | n=6 |
| 3 | Echo_lesion_: mixed | n=12 | Echo_lesion_: mixed | Number_lesion_: single | n=8 | Size_lesion_: large | Number_lesion_: single | Shape_lesion_: round | n=4 |
| 4 | Shape_lesion_: round | n=11 | Echo_lesion_: mixed | Shape_lesion_: round | n=8 | Echo_lesion_: mixed | Number_lesion_: single | Shape_lesion_: round | n=4 |
| 5 | Echo_lesion_: hypo | n=6 | Size_lesion_: large | Shape_lesion_: round | n=7 | Size_lesion_: large | Echo_lesion_: mixed | Size_organ_: enlarged | n=3 |
| 6 | Number_lesion_: multiple | n=5 | Size_lesion_: large | Shape_lesion_: round | n=7 | Size_lesion_: large | Echo_lesion_: mixed | Vasc.: decreased | n=3 |
| 7 | Margin_lesion_: ill-defined | n=5 | Echo_lesion_: mixed | Size_organ_: enlarged | n=5 | Size_lesion_: large | Number_lesion_: single | Margin_lesion_: ill-defined | n=3 |
| 8 | Size_organ_: enlarged | n=5 | Echo_lesion_: hypo | Number_lesion_: single | n=4 | Size_lesion_: large | Number_lesion_: single | Vasc.: decreased | n=3 |
| 9 | Vasc.: decreased | n=5 | Number_lesion_: single | Margin_lesion_: ill-defined | n=4 | Echo_lesion_: mixed | Number_lesion_: multiple | Shape_lesion_: round | n=3 |
| 10 |  | | Number_lesion_: single | Vasc.: decreased | n=4 | Echo_lesion_: mixed | Number_lesion_: single | Size_organ_: enlarged | n=3 |
| **Pleura**: No. Papers: n=12; No. Descriptions: n=18 | | | | | | | | | |
| 1 | Pleura_fluid_: present | n=16 | Pleura_fluid_: present | Structure_fluid_: strands | n=9 | Pleura_fluid_: present | Echo_fluid_: mixed | Structure_fluid_: strands | n=4 |
| 2 | Structure_fluid_: strands | n=9 | Pleura_fluid_: present | Echo_fluid_: mixed | n=6 | Pleura_fluid_: present | Echo_fluid_: hypo | Structure_fluid_: strands | n=3 |
| 3 | Echo_fluid_: mixed | n=6 | Pleura_fluid_: present | Echo_fluid_: hypo | n=5 |  | | | |
| 4 | Echo_fluid_: hypo | n=4 | Echo_fluid_: mixed | Structure_fluid_: strands | n=4 |  |  |  |  |
| 5 | Pleura: thickening | n=3 | Echo_fluid_: hypo | Structure_fluid_: strands | n=3 |  |  |  |  |
| **Prostate and seminal gland**: No. Papers: n=8; No. Descriptions: n=11 | | | | | | | | | |
| 1 | Number_lesion_: single | n=8 | Number_lesion_: single | Pro/sem: prostate | n=6 | Echo_lesion_: hypo | Number_lesion_: single | Pro/sem: prostate | n=4 |
| 2 | Echo_lesion_: hypo | n=7 | Echo_lesion_: hypo | Number_lesion_: single | n=5 | Echo_lesion_: hypo | Number_lesion_: single | Shape_lesion_: round | n=3 |
| 3 | Pro/sem: prostate | n=7 | Echo_lesion_: hypo | Pro/sem: prostate | n=5 | Echo_lesion_: hypo | Shape_lesion_: round | Pro/sem: prostate | n=3 |
| 4 | Echo_lesion_: mixed | n=4 | Number_lesion_: single | Shape_lesion_: round | n=4 | Number_lesion_: single | Shape_lesion_: round | Pro/sem: prostate | n=3 |
| 5 | Shape_lesion_: round | n=4 | Size_lesion_: >1cm | Number_lesion_: single | n=3 |  | | | |
| 6 | Size_organ_: enlarged | n=4 | Echo_lesion_: mixed | Number_lesion_: single | n=3 |  |  |  |  |
| 7 | Size_lesion_: >1cm | n=3 | Echo_lesion_: hypo | Shape_lesion_: round | n=3 |  |  |  |  |
| 8 |  | | Shape_lesion_: round | Pro/sem: prostate | n=3 |  |  |  |  |
| **Bones**: No. Papers: n=10; No. Descriptions: n=12 | | | | | | | | | |
| 1 | Number_lesion_: single | n=10 | Number_lesion_: single | Organ_other_: continuous | n=7 | Echo_lesion_: mixed | Number_lesion_: single | Organ_other_: continuous | n=4 |
| 2 | Organ_other_: continuous | n=9 | Echo_lesion_: mixed | Number_lesion_: single | n=6 | Echo_lesion_: hypo | Number_lesion_: single | Organ_other_: continuous | n=3 |
| 3 | Echo_lesion_: mixed | n=7 | Echo_lesion_: mixed | Organ_other_: continuous | n=5 |  | | | |
| 4 | Echo_lesion_: hypo | n=4 | Echo_lesion_: hypo | Number_lesion_: single | n=4 |  |  |  |  |
| 5 | Margin_lesion_: ill-defined | n=3 | Echo_lesion_: hypo | Organ_other_: continuous | n=3 |  |  |  |  |
| 6 |  | | Margin_lesion_: ill-defined | Organ_other_: continuous | n=3 |  |  |  |  |
| **Myocardium**: No. Papers: n=9 No. Descriptions: n=9 | | | | | | | | | |
| 1 | Size_lesion_: large | n=7 | Size_lesion_: large | Number_lesion_: single | n=6 | Size_lesion_: large | Number_lesion_: single | Surround.: endo/valve | n=4 |
| 2 | Number_lesion_: single | n=6 | Size_lesion_: large | Surround.: endo/valve | n=5 | Size_lesion_: large | Echo_lesion_: mixed | Number_lesion_: single | n=3 |
| 3 | Surround.: endo/valve | n=6 | Number_lesion_: single | Surround.: endo/valve | n=4 | Size_lesion_: large | Number_lesion_: single | Margin_lesion_: well-defined | n=3 |
| 4 | Echo_lesion_: mixed | n=4 | Size_lesion_: large | Echo_lesion_: mixed | n=3 |  | | | |
| 5 | Margin_lesion_: well-defined | n=4 | Size_lesion_: large | Margin_lesion_: well-defined | n=3 |  |  |  |  |
| 6 | Echo_lesion_: hyper | n=3 | Echo_lesion_: mixed | Number_lesion_: single | n=3 |  |  |  |  |
| 7 | Myo.: hypertrophy | n=3 | Echo_lesion_: mixed | Surround.: endo/valve | n=3 |  |  |  |  |
| 8 |  | | Number_lesion_: single | Margin_lesion_: well-defined | n=3 |  |  |  |  |
| 9 |  |  | Surround.: endo/valve | Myo.: hypertrophy | n=3 |  |  |  |  |
| **Ovaries**: No. Papers: n=8; No. Descriptions: n=9 | | | | | | | | | |
| 1 | Echo_lesion_: mixed | n=9 | Echo_lesion_: mixed | Number_lesion_: single | n=9 | Location_lesion_: localized | Echo_lesion_: mixed | Number_lesion_: single | n=7 |
| 2 | Number_lesion_: single | n=9 | Location_lesion_: localized | Echo_lesion_: mixed | n=7 | Echo_lesion_: mixed | Number_lesion_: single | Margin_lesion_: ill-defined | n=4 |
| 3 | Location_lesion_: localized | n=7 | Location_lesion_: localized | Number_lesion_: single | n=7 | Location_lesion_: localized | Echo_lesion_: mixed | Margin_lesion_: ill-defined | n=3 |
| 4 | Margin_lesion_: ill-defined | n=4 | Echo_lesion_: mixed | Margin_lesion_: ill-defined | n=4 | Location_lesion_: localized | Number_lesion_: single | Margin_lesion_: ill-defined | n=3 |
| 5 |  | | Number_lesion_: single | Margin_lesion_: ill-defined | n=4 |  | | | |
| 6 |  |  | Location_lesion_: localized | Margin_lesion_: ill-defined | n=3 |  |  |  |  |
| **Uterus**: No. Papers: n=6; No. Descriptions: n=8 | | | | | | | | | |
| 1 | Endo.line: irregular | n=4 |  | | |  | | | |
| 2 | Endo.thick.: thickened | n=2 |  |  |  |  |  |  |  |
| 3 | Echo_endometrium_: mixed | n=2 |  |  |  |  |  |  |  |
| 4 | Number_lesion_: single | n=2 |  |  |  |  |  |  |  |
| 5 | Size_lesion_: large | n=2 |  |  |  |  |  |  |  |
| 6 | Organ_other_: continuous | n=2 |  |  |  |  |  |  |  |
| **Eyes**: No. Papers: n=7; No. Descriptions: n=8 | | | | | | | | | |
| 1 | Location_lesion_: bulbus wall | n=7 | Location_lesion_: bulbus wall | Area_path_: local | n=5 | Retina: detached | Location_lesion_: bulbus wall | Area_path_: local | n=3 |
| 2 | Area_path_: local | n=6 | Retina: detached | Area_path_: local | n=4 |  | | | |
| 3 | Retina: detached | n=4 | Retina: detached | Location_lesion_: bulbus wall | n=3 |  |  |  |  |
| 4 | Sub-tenon: fluid | n=3 | Location_lesion_: bulbus wall | Sub-tenon: fluid | n=3 |  |  |  |  |
| 5 | Reflectivity: low | n=3 | Location_lesion_: bulbus wall | Reflectivity: low | n=3 |  |  |  |  |

**Legend for Table 2: variable abbreviations and explanation, values in brackets**

Number: number of aspect described (single, multiple: multiple, number not specified)

Number_ln_: number of lymph nodes

Number_lesion_: number of lesions

Number_cons_: number of lung consolidations

Echo: echogenicity (Hypo: hypoechoic, Hyper: hyperechoic, Mixed: both hyper- and hypoechoic aspects)

Echo_ln_: Echogenicity of lymph nodes

Echo_organ_: echogenicity changes of affected organ (For kidney: strata-loss: loss of corticomedullary stratification)

Echo_wall_: echogenicity of pathologic wall parts

Echo_lesion_: echogenicity of lesions

Echo_ascites_: Echogenicity of peritoneal fluid (Stranding: with stranding, Hypo: hypoechoic, Stranding+: stranding and organized material)

Echo_cons_: echogenicity of lung consolidation (mixed: hypo-/hyperechoic aspects, air bronchogram possible)

Echo_endometrium_: echogenicity of endometrium

Echo_PNodules_: Echogenicity of peritoneal nodules

Echo_fluid_: Echogenicity of pericardial fluid

Echo_perimass_: echogenicity of intrapericardial mass

Echo_Peritoneum_: Echogenicity of thickened peritoneum or omentum

Size: size of aspect described

Size_organ_: Size of organ (enlarged (lymph nodes >1.5cm; spleen >11cm)

Size_cons_: size of consolidation

Size_lesion_: size of lesions (testis, epididymis, thyroid, myocardium, uterus: <1cm small, >1cm large; breast, ovaries: <5cm small; >5cm large; spleen, kidney, bone, liver: <1cm small; 1-5cm medium; >5cm large; pancreas: <2cm small; >2cm large; esophagus: <1.5cm small; >1.5cm large)

Shape: shape of aspect described (round: round or oval or nodular; irregular: irregular or no clear shape; lobulated: lobulated or landscape-like)

Shape_ln_: shape of lymph node

Shape_lesion_: shape of lesions

Shape_Mass_: Shape of mass

Shape_cons_: shape of lung consolidations (round: round or oval; shred: no clear shape with shredded or irregular posterior border; mixed: no uniform pattern, variable shapes)

Margin: margin quality of aspect described

Margin_ln_: Margin quality of lymph node (ill-defined: ill-defined, matting or bulking)

Margin_lesion_: margin quality of lesions (for bones: ill-defined includes bone erosions)

Margin_capsule_: Kidney capsule margin quality (ill-defined: ill-defined or irregular)

Location: location of aspect described

Location_lesion_: location of lesions (Localized: localized or limited to one area; dissem.: disseminated, not limited, multiple areas affected; for prostate and seminal glands: periph.: peripheral prostate areas)

Location_ln_: location of lymph nodes in the abdomen (mesent.: in the mesentery; panc: para-pancreatic; liver: liver hilum; diss.: disseminated or >1 area)

Location_PChanges_: Location of peritoneal changes (omentum: only omentum)

Organ_other_: other organs affected (general: continuous: lesion extends to surrounding tissues, e.g. muscle, skin etc.; proximity: proximal organs affected e.g., neighboring organs; distant: distant organs affected, no direct neighborhood) (For intestines: abd.ln: abdominal lymphadenopathy; for bones: continuous: involved surrounding tissues, e.g. joints; for uterus: continuous: involved surrounding structures, e.g. tubes)

Fluid-variables: refers to cavitary fluids as specified (large: large amount; present: present, amount not specified)

Peri_fluid_: pericardial fluid

Pleura_fluid_: pleural fluid

Amount_Pl.fluid_/Amount_ascites_ amount of pleural/peritoneal fluid (small: small or loculated)

Structure_fluid_: structured content of pericardial fluid (strands: stranding or linear fibrinous elements; mass: mass-like organized lesion)

Thoracic and lung variables:

P.line: pleural line pathologies (irregular: irregular line; gap: interrupted pleural line adjacent to consolidation)

Vertical: vertical lung ultrasound artefacts

Pericardium: changes in the pericardium itself (thickening: generalized laminar thickening; nodular: focal or nodular thickening)

Myo: myocardium

Med.ln.: mediastinal or para-esophageal lymph nodes (continuous: direct connection with esophagus; present: lymph nodes present, direct contact with esophagus not specified)

Intranodal: Intranodal lesions (necro: hypo-/anechoic necrosis zone; hilum loss; hyper: hyperechoic lesions, including calcifications)

General other variables:

Post. phen.: posterior phenomena (enh.: enhancement)

Vasc.: vascularity (decreased; increased; present: normal or preserved; mixed: areas with hypo- and areas with hypervascularity; for lymph nodes: distribution: abnormal vessel distribution)

Vasc.pattern: distribution pattern of vessels (mixed: flow in both hilar and cortical structures)

Surround.: surrounding tissue changes (general: hyper: hyperechoic change; hypo: hypoechoic change; fluid: fluid surrounding the organ) (for intrathoracic lymph nodes: matting: matting of surrounding lymph nodes; for myocardium: endo/valve: endocardium and/or valves affected)

Urinary tract and kidney variables:

Urothelium: changes in the urothelium (thickening: thickening of the urothelium)

U.tract: Changes in the urinary tract

Abdominal and gastrointestinal variables:

Peritoneum: Peritoneal changes (nodular: nodular thickening; thickening: generalized thickening)

Intraperitoneal: Additional intra-peritoneal findings (mass; matting: Bowel matting)

Wall_thick_: in gastrointestinal organs thickness of wall

Wall_location_: location of lesions/pathology in gastrointestinal organ wall (circum: circumferential; focal: focal/non-circumferential)

Wall_archi_: wall architecture (disrupted)

Length: length of intestinal wall pathology (long: long or >5cm)

Passage: regards intestinal passage pathologies

Reproductive organ variables:

Pro/sem: prostate or seminal glands affected (prostate: prostate only; seminal: seminal glands only; prostate+sem.: both prostate and seminal glands)

Endo.line: endometrial lining changes (Irregular: irregularity, e.g. nodular)

Endo.thick: thickness of endometrium

Eye-related variables:

Retina: retinal changes (detached: retinal detachment)

Reflectivity: internal reflectivity

Sub-tenon: changes in the sub-tenon space

Area_path_: extent of pathologies (local: localized or singular lesions)

Bulbus wall: choroidal or scleral or retinal location of pathology

* n=8 for five 2tuple combinations: [Echo_lesion_: mixed + Number_lesion_: single]; [Echo_lesion_: hypo + Surround.: fluid]; [Shape_lesion_: round/oval + Size_organ_: enlarged]; [Shape_lesion_: round/oval + Organ_other_: proximity]; [Surround.: fluid + Organ_other_: proximity]

** n=6 for nine 2tuple combinations: [Size_lesion_: large + Location_lesion_: localized + Echo_lesion_: mixed]; [Size_lesion_: large + Location_lesion_: localized + Number_lesion_: single]; [Size_lesion_: small + Echo_lesion_: hypo + Number_lesion_: multiple]; [Size_lesion_: large + Number_lesion_: single + Echo_lesion_: mixed]; [Echo_lesion_: hypo + Number_lesion_: multiple + Echo_lesion_: mixed]; [Echo_lesion_: hypo + Number_lesion_: multiple + Vasc.: increased]; [Echo_lesion_: hypo + Shape_lesion_: round/oval + Size_organ_: enlarged]; [Echo_lesion_: hypo + Shape_lesion_: round/oval + Organ_other_: proximity]; [Echo_lesion_: hypo + Size_organ_: enlarged + Organ_other_: proximity]

**c) Supplement Table S3a (sensitivity analysis only confirmed TB)**

Limited to features with at least n=4 descriptions.

| **Breast;** No. Papers: n= 14/25; No. Descriptions: n=28/45 | | | | | | | | | |
| --- | --- | --- | --- | --- | --- | --- | --- | --- | --- |
|  | ***Single feature*** | | ***Two-feature combinations*** | | | ***Three-feature combinations*** | | | |
| 1 | Number_lesion_: single | n=21 | Number_lesion_: single | Echo_lesion_: mixed | n=15 | Echo_lesion_: mixed | Number_lesion_: single | Margin_lesion_: ill-defined | n=9 |
| 2 | Echo_lesion_: mixed | n=19 | Echo_lesion_: mixed | Margin_lesion_: ill-defined | n=10 | Echo_lesion_: mixed | Number_lesion_: single | Post. phen: enh. | n=7 |
| 3 | Margin_lesion_: ill-defined | n=12 | Margin_lesion_: ill-defined | Number_lesion_: single | n=10 | Echo_lesion_: mixed | Margin_lesion_: ill-defined | Post. phen: enh. | n=6 |
| 4 | Post. phen: enh. | n=11 | Echo_lesion_: mixed | Post. phen: enh. | n=9 | Echo_lesion_: mixed | Number_lesion_: single | Organ_other_: continuous | n=5 |
| 5 | Shape_lesion_: round/oval | n=9 | Number_lesion_: single | Post. phen: enh. | n=8 | Margin_lesion_: ill-defined | Number_lesion_: single | Post. phen: enh. | n=5 |
| 6 | Location_lesion_: localized | n=8 | Number_lesion_: single | Location_lesion_: localized | n=7 | Number_lesion_: single | Echo_lesion_: mixed | Location_lesion_: localized | n=4 |
| 7 | Organ_other_: continuous | n=8 | Number_lesion_: single | Shape_lesion_: round/oval | n=7 | Number_lesion_: single | Shape_lesion_: round/oval | Echo_lesion_: mixed | n=4 |
| 8 | Echo_lesion_: hypo | n=7 | Echo_lesion_: hypo | Number_lesion_: single | n=6 |  |  |  | *n=3(10x)* |
| 9 | Size_lesion_: large | n=4 | Echo_lesion_: mixed | Organ_other_: continuous | n=6 |  | | | |
| 10 | Margin_lesion_: well-defined | n=4 | Number_lesion_: single | Organ_other_: continuous | n=6 |  |  |  |  |
| 11 | Vasc.: decreased | n=4 | Margin_lesion_: ill-defined | Post. phen: enh. | n=6 |  |  |  |  |
| 12 | Calcification: present | n=4 | Echo_lesion_: mixed | Shape_lesion_: round/oval | n=5 |  |  |  |  |
| 13 |  | n=3 (2x) |  |  | n=4 (5x) |  |  |  |  |
| **Liver;** No. Papers: n= 18/30; No. descriptions: n=23/ 40 | | | | | | | | | |
| 1 | Number_lesion_: multiple | n=16 | Echo_lesion_: hypo | Number_lesion_: multiple | n=11 | Size_lesion_: small | Echo_lesion_: hypo | Number_lesion_: multiple | n=7 |
| 2 | Echo_lesion_: hypo | n=14 | Location_lesion_: dissem. | Number_lesion_: multiple | n=9 | Location_lesion_: dissem. | Echo_lesion_: hypo | Number_lesion_: multiple | n=7 |
| 3 | Location_lesion_: dissem. | n=9 | Size_lesion_: small | Echo_lesion_: hypo | n=7 | Size_lesion_: small | Location_lesion_: dissem. | Echo_lesion_: hypo | n=4 |
| 4 | Size_lesion_: small | n=7 | Size_lesion_: small | Number_lesion_: multiple | n=7 | Size_lesion_: small | Location_lesion_: dissem. | Number_lesion_: multiple | n=4 |
| 5 | Echo_lesion_: mixed | n=7 | Location_lesion_: dissem. | Echo_lesion_: hypo | n=7 | Location_lesion_: dissem. | Echo_lesion_: hypo | Size_Organ_: enlarged | n=4 |
| 6 | Shape_lesion_: round/oval | n=7 | Shape_lesion_: round/oval | Echo_lesion_: hypo | n=5 | Location_lesion_: dissem. | Number_lesion_: multiple | Shape_lesion_: round/oval | n=4 |
| 7 | Location_lesion_: localized | n=6 | Shape_lesion_: round/oval | Number_lesion_: multiple | n=5 | Location_lesion_: dissem. | Number_lesion_: multiple | Size_Organ_: enlarged | n=4 |
| 8 | Margin_lesion_: ill-defined | n=5 |  |  | n=4 (8x) | Echo_lesion_: hypo | Number_lesion_: multiple | Size_Organ_: enlarged | n=4 |
| **Pancreas;** No. Papers: n= 21/34; No. Descritpions: n= 23/37 | | | | | | | | | |
| 1 | Number_lesion_: single | n=18 | Number_lesion_: single | Size_lesion_: large | n=13 | Number_lesion_: single | Size_lesion_: large | Location_lesion_: localized | n=9 |
| 2 | Size_lesion_: large | n=13 | Number_lesion_: single | Location_lesion_: localized | n=13 | Number_lesion_: single | Size_lesion_: large | Echo_lesion_: mixed | n=7 |
| 3 | Location_lesion_: localized | n=13 | Location_lesion_: localized | Size_lesion_: large | n=9 | Location_lesion_: localized | Echo_lesion_: hypo | Number_lesion_: single | n=7 |
| 4 | Echolesion: hypo | n=11 | Number_lesion_: single | Echo_lesion_: hypo | n=9 | Size_lesion_: large | Echo_lesion_: hypo | Number_lesion_: single | n=6 |
| 5 | Echo_lesion_: mixed | n=9 | Echo_lesion_: mixed | Number_lesion_: single | n=9 | Number_lesion_: single | Location_lesion_: localized | Echo_lesion_: mixed | n=6 |
| 6 | Margin_lesion_: ill-defined | n=7 | Size_lesion_: large | Echo_lesion_: mixed | n=7 | Echo_lesion_: hypo | Size_lesion_: large | Location_lesion_: localized | n=5 |
| 7 | Location_lesion_: dissem. | n=5 | Location_lesion_: localized | Echo_lesion_: hypo | n=7 | Location_lesion_: localized | Margin_lesion_: ill-defined | Number_lesion_: single | n=5 |
| 8 | Margin_lesion_: well-defined | n=5 | Size_lesion_: large | Echo_lesion_: hypo | n=6 | Location_lesion_: localized | Size_lesion_: large | Echo_lesion_: mixed | n=4 |
| 9 | Shape_lesion_: lobulated | n=4 | Echo_lesion_: mixed | Location_lesion_: localized | n=6 | Size_lesion_: large | Margin_lesion_: ill-defined | Number_lesion_: single | n=4 |
| 10 | Vasc.: decreased | n=4 | Number_lesion_: single | Margin_lesion_: ill-defined | n=6 | Margin_lesion_: ill-defined | Number_lesion_: single | Echo_lesion_: mixed | N=4 |
| 11 |  | n=3 (2x) | Location_lesion_: localized | Margin_lesion_: ill-defined | n=5 |  | | | |
|  |  | |  |  | n=4(6x) |  |  |  |  |
| **Testis:** No. Papers: n= 8/21; No. Descriptions: n=9/36 | | | | | | | | | |
| 1 | Size_lesion_: >1cm large | n=6 | Size_lesion_: >1cm large | Location_lesion_: localized | n=6 | Size_lesion_: >1cm large | Location_lesion_: localized | Echo_lesion_: mixed | n=5 |
| 2 | Location_lesion_: localized | n=6 | Size_lesion_: >1cm large | Echo_lesion_: mixed | n=5 | Size_lesion_: >1cm large | Location_lesion_: localized | Number_lesion_: single | n=5 |
| 3 | Echo_lesion_: mixed | n=5 | Size_lesion_: >1cm large | Number_lesion_: single | n=5 | Size_lesion_: >1cm large | Echo_lesion_: mixed | Number_lesion_: single | n=4 |
| 4 | Number_lesion_: single | n=5 | Location_lesion_: localized | Echo_lesion_: mixed | n=5 | Location_lesion_: localized | Echo_lesion_: mixed | Number_lesion_: single | n=4 |
| 5 | Vasc.: increased | n=5 | Location_lesion_: localized | Number_lesion_: single | n=5 |  |  |  | n=3 (8x) |
| 6 | Organ_other_: proximity | n=5 | Echo_lesion_: mixed | Number_lesion_: single | n=4 |  | | | |
| 7 | Echolesion: hypo | n=4 | Vasc.: increased | Organ_other_: proximity | n=4 |  |  |  |  |
| 8 | Number_lesion_: multiple | n=4 |  |  | n=3 (13x) |  |  |  |  |
| 9 | Size_organ_: enlarged | n=4 |  | | |  |  |  |  |
| **Epididymis:** No. papers: n= 9/22; No. Descriptions: n=12/35 | | | | | | | | | |
| 1 | Organ_other_: proximity | n=7 | Location_lesion_: localized | Organ_other_: proximity | n=4 |  |  |  | *n=2(9x)* |
| 2 | Location_lesion_: localized | n=6 | Organ_other_: proximity | Size_organ_: enlarged | n=4 |  | | | |
| 3 | Size_organ_: enlarged | n=6 | Vasc.: increased | Organ_other_: proximity | n=4 |  |  |  |  |
| 4 | Echo_organ_: hypo | n=4 |  |  | n=3 (4x) |  |  |  |  |
| 5 | Vasc.: increased | n=4 |  | | |  |  |  |  |
| **Spleen:** No. Papers: n= 15/24; No. Descriptions: n=18/31 | | | | | | | | | |
| 1 | Echo_lesion_: hypo | n=15 | Echo_lesion_: hypo | Number_lesion_: multiple | n=13 | Size_lesion_: small | Echo_lesion_: hypo | Number_lesion_: multiple | n=7 |
| 2 | Number_lesion_: multiple | n=14 | Size_lesion_: small | Echo_lesion_: hypo | n=8 | Location_lesion_: dissem. | Echo_lesion_: hypo | Number_lesion_: multiple | n=5 |
| 3 | Size_lesion_: small | n=8 | Size_lesion_: small | Number_lesion_: multiple | n=7 | Echo_lesion_: hypo | Location_lesion_: dissem. | Size_lesion_: small | n=4 |
| 4 | Location_lesion_: dissem. | n=7 | Location_lesion_: dissem. | Echo_lesion_: hypo | n=6 | Size_lesion_: small | Number_lesion_: multiple | Location_lesion_: dissem. | n=4 |
| 5 | Margin_lesion_: well-defined | n=4 | Location_lesion_: dissem. | Number_lesion_: multiple | n=6 | Echo_lesion_: hypo | Number_lesion_: multiple | Size_organ_: enlarged | n=4 |
| 6 | Size_organ_: enlarged | n=4 | Size_lesion_: small | Number_lesion_: multiple | n=4 | Echo_lesion_: hypo | Number_lesion_: multiple | Organ_other_: distant | n=4 |
| 7 | Organ_other_: distant | n=4 | Echo_lesion_: hypo | Size_organ_: enlarged | n=4 |  |  |  | n=3 (4x) |
| 8 |  | n=3 (4x) | Echo_lesion_: hypo | Organ_other_: distant | n=4 |  | | | |
| 9 |  | | Number_lesion_: multiple | Size_organ_: enlarged | n=4 |  |  |  |  |
| 10 |  |  | Number_lesion_: multiple | Organ_other_: distant | n=4 |  |  |  |  |
| **Peripheral lymph nodes;** No. Papers: n= 8/24 No. Descriptions: n=13/36 | | | | | | | | | |
| 1 | Echo_ln_: mixed | n=8 | Echo_ln_: mixed | Number_ln_: multiple | n=6 |  |  |  | *n=3(2x)* |
| 2 | Intranodal: necro | n=7 | Echo_ln_: mixed | Intranodal: necro | n=5 |  | | | |
| 3 | Number_ln_: multiple | n=6 |  |  | n=3 (2x) |  |  |  |  |
| 4 | Number_ln_: single | n=4 |  | | |  |  |  |  |
| 5 | Shape_ln_: rounded | n=4 |  |  |  |  |  |  |  |
| 6 | Vasc.: preseved | n=4 |  |  |  |  |  |  |  |
| **Peritoneum and omentum;** No. Papers: n= 9/19 No. Descriptions: n=14/35 | | | | | | | | | |
| 1 | Ascites: present | n=8 | Ascites: present | Echo_ascites_: stranding | n=4 |  |  |  | *n=2 (4x)* |
| 2 | Peritoneum: thickening | n=5 |  |  | n=3 (1x) |  | | | |
| 3 | Location_PChanges_: Omentum | n=5 |  | | |  |  |  |  |
| 4 | Echo_ascites_: stranding | n=4 |  |  |  |  |  |  |  |
| 5 | Peritoneum: Nodular | n=4 |  |  |  |  |  |  |  |
| **Kidney:** No. Papers: n= 3/10; No. Descriptions: n=4/28 | | | | | | | | | |
| 1 |  | *n=2 (4x)* |  |  | *n=2 (1x)* |  |  |  | *n=1 (25x)* |
| **Pericardium:** No. Papers: n= 14/21; No. Descriptions: n=14/26 | | | | | | | | | |
| 1 | Peri_fluid_: large | n=10 | Peri_fluid_: large | Structure_fluid_: strands | n=7 |  |  |  | *n=3 (1x)* |
| 2 | Structure_fluid_: strands | n=7 | Peri_fluid_: large | Echo_fluid_: mixed | n=5 |  | | | |
| 3 | Echo_fluid_: mixed | n=5 |  |  | n=3 (3x) |  |  |  |  |
| 4 | Structure_fluid_: mass | n=4 |  | | |  |  |  |  |
| 5 | Pericardium: thickening | n=4 |  |  |  |  |  |  |  |
| **Esophagus**: No. Papers: n=8/15; No. Descriptions: n=11/26 | | | | | | | | | |
| 1 | Wall_archi_: disrupted | n=7 | Calcification: present | Med.ln.: continuous | n=4 |  |  |  | *n=2 (4x)* |
| 2 | Wall_thick_: thickened | n=6 |  |  | *n=3 (5x)* |  | | | |
| 3 | Med.ln.: continuous | n=5 |  | | |  |  |  |  |
| 4 | Number_lesion_: single | n=4 |  |  |  |  |  |  |  |
| 5 | Calcification: present | n=4 |  |  |  |  |  |  |  |
| **Intrathoracic lymph nodes**: No. Papers: n=12/20; No. Descriptions: n=17/26 | | | | | | | | | |
| 1 | Number_ln_: multiple | n=11 | Number_ln_: multiple | Sizeorgan: enlarged | n=8 | Size_organ_: enlarged | Echo_ln_: mixed | Number_ln_: multiple | n=5 |
| 2 | Echo_ln_: mixed | n=10 | Size_organ_: enlarged | Margin_ln_: ill-defined | n=6 | Size_organ_: enlarged | Number_ln_: multiple | Margin_ln_: ill-defined | n=5 |
| 3 | Organ_other_: esophagus | n=10 | Size_organ_: enlarged | Organ_other_: esophagus | n=6 | Size_organ_: enlarged | Organ_other_: esophagus | Number_ln_: multiple | n=5 |
| 4 | Size_organ_: enlarged | n=9 | Echo_ln_: mixed | Number_ln_: multiple | n=6 | Size_organ_: enlarged | Echo_ln_: mixed | Margin_ln_: ill-defined | n=4 |
| 5 | Margin_ln_: ill-defined | n=8 | Echo_ln_: mixed | Organ_other_: esophagus | n=6 | Size_organ_: enlarged | Margin_ln_: ill-defined | Intranodal: hyper | n=4 |
| 6 | Intranodal: hyper | n=6 | Number_ln_: multiple | Margin_ln_: ill-defined | n=6 | Size_organ_: enlarged | Margin_ln_: ill-defined | Organ_other_: esophagus | n=4 |
| 7 | Number_ln_: single | n=5 | Number_ln_: multiple | Organ_other_: esophagus | n=6 | Size_organ_: enlarged | Organ_other_: esophagus | Intranodal: hyper | n=4 |
| 8 | Surround.: matting | n=5 | Size_organ_: enlarged | Echo_ln_: mixed | n=5 | Number_ln_: multiple | Echo_ln_: mixed | Margin_ln_: ill-defined | n=4 |
| 9 |  | n=3 (2x) | Intranodal: hyper | Echo_ln_: mixed | n=5 | Number_ln_: multiple | Intranodal: hyper | Echo_ln_: mixed | n=4 |
| 10 |  | | Organ_other_: esophagus | Intranodal: hyper | n=5 | Echo_ln_: mixed | Intranodal: hyper | Organ_other_: esophagus | n=4 |
| 11 |  |  |  |  | n=4 (9x) | Margin_ln_: ill-defined | Intranodal: hyper | Organ_other_: esophagus | n=4 |
| **Abdominal lymph nodes**: No. Papers: n=12/19; No. Descriptions: n=15/23 | | | | | | | | | |
| 1 | Size_organ_: enlarged | n=13 | Size_organ_: enlarged | Echo_ln_: hypo | n=8 | Size_organ_: enlarged | Echo_ln_: hypo | Shape_ln_: rounded | n=5 |
| 2 | Echo_ln_: hypo | n=8 | Size_organ_: enlarged | Number_ln_: multiple | n=6 | Size_organ_: enlarged | Number_ln_: multiple | Shape_ln_: rounded | n=5 |
| 3 | Number_ln_: multiple | n=7 | Size_organ_: enlarged | Shape_ln_: rounded | n=6 | Size_organ_: enlarged | Number_ln_: multiple | Echo_ln_: hypo | n=4 |
| 4 | Shape_ln_: rounded | n=6 | Size_organ_: enlarged | Number_ln_: single | n=5 | Echo_ln_: hypo | Number_ln_: multiple | Shape_ln_: rounded | n=4 |
| 5 | Number_ln_: single | n=5 | Echo_ln_: hypo | Shape_ln_: rounded | n=5 |  |  |  | *n=3 (3x)* |
| 6 | Location_ln_: panc. | n=4 | Number_ln_: multiple | Shape_ln_: rounded | n=5 |  | | | |
| 7 | Intranodal: necro | n=4 | Echo_ln_: hypo | Number_ln_: multiple | n=4 |  |  |  |  |
| **Lung**: No. Papers: n=9/15 No. Descriptions: n=18/24 | | | | | | | | | |
| 1 | Echo_cons_: hypo | n=6 | Echo_cons_: hypo | Shape_cons_: round | n=4 |  |  |  | *n=3 (4x)* |
| 2 | Shape_cons_: round | n=5 |  |  | *n=3 (8x)* | . | | | |
| 3 | Post. phen: enh. | n=5 |  | | |  |  |  |  |
| 4 | Number_cons_: multiple | n=5 |  |  |  |  |  |  |  |
| 5 | Size_cons_: <1cm | n=4 |  |  |  |  |  |  |  |
| 6 | Size_cons_: >1cm | n=4 |  |  |  |  |  |  |  |
| 7 | P.line: irregular | n=4 |  |  |  |  |  |  |  |
| 8 | Vertical: B-lines | n=4 |  |  |  |  |  |  |  |
| 9 | Location_lesion_: multiple | n=4 |  |  |  |  |  |  |  |
| **Intestines**: No. Papers: n=3/14; No. Descriptions: n=7/21 | | | | | | | | | |
| 1 | Wall_thick_: thickening | n=5 |  |  | *n=2 (5x)* | *n=2 (1x)* | | | |
| 2 |  | *n=3 (1x)* |  | | |  | | | |
| **Thyroid**: No. Papers: n=9/15; No. Descriptions: n=11/19 | | | | | | | | | |
| 1 | Number_lesion_: single | n=7 | Size_lesion_: large | Echo_lesion_: mixed | n=4 |  |  |  | *n=3 (1x)* |
| 2 | Size_lesion_: large | n=6 | Size_lesion_: large | Number_lesion_: single | n=4 |  | | | |
| 3 | Echo_lesion_: mixed | n=6 | Echo_lesion_: mixed | Number_lesion_: single | n=4 |  |  |  |  |
| 4 | Echo_lesion_: hypo | n=5 | Echo_lesion_: mixed | Shape_lesion_: round | n=4 |  |  |  |  |
| 5 | Shape_lesion_: round | n=5 |  |  | *n=3 (3x)* |  |  |  |  |
| 6 | Number_lesion_: multiple | n=4 |  | | |  |  |  |  |
| 7 |  | *n=3 (2x)* |  |  |  |  |  |  |  |
| **Pleura**: No. Papers: n=7/12; No. Descriptions: n=8/18 | | | | | | | | | |
| 1 | Pleura_fluid_: present | n=6 | Pleura_fluid_: present | Structure_fluid_: strands | n=5 |  |  |  | *n=2 (1x)* |
| 2 | Structure_fluid_: strands | n=5 |  |  | *n=2 (2x)* |  | | | |
| 3 |  | *n=2 (2x)* |  | | |  |  |  |  |
| **Prostate and seminal gland**: No. Papers: n=4/8; No. Descriptions: n=4/11 | | | | | | | | | |
| 1 |  | *n=3 (3x)* |  |  | *n=3 (1x)* |  |  |  | *n=2 (2x)* |
| **Bones**: No. Papers: n=6/10; No. Descriptions: n=6/12 | | | | | | | | | |
| 1 | Number_lesion_: single | n=5 | Number_lesion_: single | Echo_lesion_: mixed | n=4 |  |  |  | *n=2 (2x)* |
| 2 | Organ_other_: continuous | n=5 |  |  | *n=3 (1x)* |  | | | |
| 3 |  | *n=3 (1x)* |  | | |  |  |  |  |
| **Myocardium**: No. Papers: n=4/9 No. Descriptions: n=4/9 | | | | | | | | | |
| 1 |  | *n=3 (4x)* |  |  | *n=3 (2x)* |  |  |  | *n=2 (5x)* |
| **Ovaries**: No. Papers: n=1/8; No. Descriptions: n=1/9 | | | | | | | | | |
| 1 |  | *n=1 (5x)* |  |  | *n=1 (10x)* |  |  |  | *n=1 (10x)* |
| **Uterus**: No. Papers: n=3/6; No. Descriptions: n=3/8 | | | | | | | | | |
| 1 |  | *n=2 (1x)* | *n=1 (12x)* | | | *n=1 (10x)* | | | |
| **Eyes**: No. Papers: n=3/7; No. Descriptions: n=4/8 | | | | | | | | | |
| 1 |  | *n=3 (3x)* |  |  | *n=3 (1x)* |  |  |  | *n=2 (1x)* |

**d) Supplement Table S3a (sensitivity analysis only confirmed TB and representative)**

Limited to articles with n=4 descriptions or more, if less, this is indicated.

| **Breast;** No. Papers: n= 0/25; No. Descriptions: n=0/45 | | | | | | | | | |
| --- | --- | --- | --- | --- | --- | --- | --- | --- | --- |
|  | ***Single feature*** | | ***Two-feature combinations*** | | | ***Three-feature combinations*** | | | |
| 1 |  |  |  |  |  |  |  |  |  |
| **Liver;** No. Papers: n= 2/30; No. descriptions: n=2/ 40 | | | | | | | | | |
| 1 | *Number_lesion_: multiple* | *n=2 (1x)* |  |  | *n=1 (18x)* |  |  |  | *n=1 (21x)* |
| **Pancreas;** No. Papers: n= 0/34; No. Descritpions: n= 0/37 | | | | | | | | | |
| 1 |  |  |  |  |  |  |  |  |  |
| **Testis:** No. Papers: n= 1/21; No. Descriptions: n=1/36 | | | | | | | | | |
| 1 | *Size_lesion_: >1cm large* | *n=1 (7x)* | *Size_lesion_: >1cm large* | *Location_lesion_: localized* | *n=1 (21x)* | *Size_lesion_: >1cm large* | *Location_lesion_: localized* | *Echo_lesion_: mixed* | *n=1 (25x)* |
| **Epididymis:** No. papers: n= 1/22; No. Descriptions: n=1/35 | | | | | | | | | |
| 1 |  | *n= 1 (4x)* |  |  | *n=1 (6x)* |  |  |  | *n=1 (4x)* |
| **Spleen:** No. Papers: n= 1/24; No. Descriptions: n=1/31 | | | | | | | | | |
| 1 |  | *n=1 (3x)* |  |  | *n=1 (3x)* |  |  |  | *n=1 (1x)* |
| **Peripheral lymph nodes;** No. Papers: n= 2/24 No. Descriptions: n=11/36 | | | | | | | | | |
| 1 | *Echo_ln_: mixed* | *n=1 (4x)* |  |  | *n=1 (6x)* |  |  |  | *n=1 (4x)* |
| **Peritoneum and omentum;** No. Papers: n= 1/19 No. Descriptions: n=5/35 | | | | | | | | | |
| 1 | Location_PChanges_: Omentum | n=5 |  |  | *n=3 (1x)* |  |  |  | *n=2 (1x)* |
| 2 | *Peritoneum: thickening* | *n=3 (1x)* |  |  |  |  |  |  |  |
| **Kidney:** No. Papers: n= 0/10; No. Descriptions: n=0/28 | | | | | | | | | |
| 1 |  |  |  |  |  |  |  |  |  |
| **Pericardium:** No. Papers: n= 2/21; No. Descriptions: n=2/26 | | | | | | | | | |
| 1 |  | *n=2 (2x)* |  |  | *n=2 (1x)* |  |  |  | *n=1 (4x)* |
| **Esophagus**: No. Papers: n=1/15; No. Descriptions: n=2/26 | | | | | | | | | |
| 1 | *Wall_archi_: disrupted* | *n=2 (1x)* |  |  | *n=1 (4x)* |  |  |  | *n=1 (1x)* |
| **Intrathoracic lymph nodes**: No. Papers: n=3/20; No. Descriptions: n=6/26 | | | | | | | | | |
| 1 | Echo_ln_: mixed | n=4 |  |  | *n=3 (2x)* |  |  |  | *n=2 (5x)* |
| 2 |  | *n=3 (4x)* |  |  |  |  |  |  |  |
| **Abdominal lymph nodes**: No. Papers: n=0/19; No. Descriptions: n=0/23 | | | | | | | | | |
| 1 |  |  |  |  |  |  |  |  |  |
| **Lung**: No. Papers: n=4/15 No. Descriptions: n=10/24 | | | | | | | | | |
| 1 | Echo_cons_: hypo | n=4 | *Echo_cons_: hypo* | *Shape_cons_: round* | *n=3 (1x)* |  |  |  | *n=2 (4x)* |
| 2 | Shape_cons_: round | n=4 |  |  |  |  |  |  |  |
| 3 |  | *n=3 (1x)* |  |  |  |  |  |  |  |
| **Intestines**: No. Papers: n=1/14; No. Descriptions: n=5/21 | | | | | | | | | |
| 1 | *Wall_thick_: thickening* | *n=3 (2x)* |  |  | *n=2 (3x)* | *n=2 (1x)* | | | |
| **Thyroid**: No. Papers: n=0/15; No. Descriptions: n=0/19 | | | | | | | | | |
| 1 |  |  |  |  |  |  |  |  |  |
| **Pleura**: No. Papers: n=0/12; No. Descriptions: n=0/18 | | | | | | | | | |
| 1 |  |  |  |  |  |  |  |  |  |
| **Prostate and seminal gland**: No. Papers: n=1/8; No. Descriptions: n=1/11 | | | | | | | | | |
| 1 |  | *n=1 (7x)* |  |  | *n=1 (21x)* |  |  |  | *n=1 (25x)* |
| **Bones**: No. Papers: n=1/10; No. Descriptions: n=1/12 | | | | | | | | | |
| 1 |  | *n=1 (5x)* | *Number_lesion_: single* | *Echo_lesion_: mixed* | *n=1 (10x)* |  |  |  | *n=1 (10x)* |
| **Myocardium**: No. Papers: n=1/9 No. Descriptions: n=1/9 | | | | | | | | | |
| 1 |  | *n=1 (5x)* |  |  | *n=1 (10x)* |  |  |  | *n=1 (10x)* |
| **Ovaries**: No. Papers: n=0/8; No. Descriptions: n=0/9 | | | | | | | | | |
| 1 |  |  |  |  |  |  |  |  |  |
| **Uterus**: No. Papers: n=0/6; No. Descriptions: n=0/8 | | | | | | | | | |
| 1 |  |  |  | | |  | | | |
| **Eyes**: No. Papers: n=0/7; No. Descriptions: n=0/8 | | | | | | | | | |
| 1 |  |  |  |  |  |  |  |  |  |
| **Breast;** No. Papers: n= 0/25; No. Descriptions: n=0/45 | | | | | | | | | |
|  | ***Single feature*** | | ***Two-feature combinations*** | | | ***Three-feature combinations*** | | | |
| 1 |  | |  | | |  | | | |
| **Liver;** No. Papers: n= 2/30; No. descriptions: n=2/ 40 | | | | | | | | | |
| 1 | *Number_lesion_: multiple* | *n=2 (1x)* |  |  | *n=1 (18x)* |  |  |  | *n=1 (21x)* |
| **Pancreas;** No. Papers: n= 0/34; No. Descritpions: n= 0/37 | | | | | | | | | |
| 1 |  | |  | | |  | | | |
| **Testis:** No. Papers: n= 1/21; No. Descriptions: n=1/36 | | | | | | | | | |
| 1 | *Size_lesion_: >1cm large* | *n=1 (7x)* | *Size_lesion_: >1cm large* | *Location_lesion_: localized* | *n=1 (21x)* | *Size_lesion_: >1cm large* | *Location_lesion_: localized* | *Echo_lesion_: mixed* | *n=1 (25x)* |
| **Epididymis:** No. papers: n= 1/22; No. Descriptions: n=1/35 | | | | | | | | | |
| 1 |  | *n= 1 (4x)* |  |  | *n=1 (6x)* |  |  |  | *n=1 (4x)* |
| **Spleen:** No. Papers: n= 1/24; No. Descriptions: n=1/31 | | | | | | | | | |
| 1 |  | *n=1 (3x)* |  |  | *n=1 (3x)* |  |  |  | *n=1 (1x)* |
| **Peripheral lymph nodes;** No. Papers: n= 2/24 No. Descriptions: n=11/36 | | | | | | | | | |
| 1 | *Echo_ln_: mixed* | *n=1 (4x)* |  |  | *n=1 (6x)* |  |  |  | *n=1 (4x)* |
| **Peritoneum and omentum;** No. Papers: n= 1/19 No. Descriptions: n=5/35 | | | | | | | | | |
| 1 | Location_PChanges_: Omentum | n=5 |  |  | *n=3 (1x)* |  |  |  | *n=2 (1x)* |
| 2 | *Peritoneum: thickening* | *n=3 (1x)* |  | | |  | | | |
| **Kidney:** No. Papers: n= 0/10; No. Descriptions: n=0/28 | | | | | | | | | |
| 1 |  | |  | | |  | | | |
| **Pericardium:** No. Papers: n= 2/21; No. Descriptions: n=2/26 | | | | | | | | | |
| 1 |  | *n=2 (2x)* |  |  | *n=2 (1x)* |  |  |  | *n=1 (4x)* |
| **Esophagus**: No. Papers: n=1/15; No. Descriptions: n=2/26 | | | | | | | | | |
| 1 | *Wall_archi_: disrupted* | *n=2 (1x)* |  |  | *n=1 (4x)* |  |  |  | *n=1 (1x)* |
| **Intrathoracic lymph nodes**: No. Papers: n=3/20; No. Descriptions: n=6/26 | | | | | | | | | |
| 1 | Echo_ln_: mixed | n=4 |  |  | *n=3 (2x)* |  |  |  | *n=2 (5x)* |
| 2 |  | *n=3 (4x)* |  | | |  | | | |
| **Abdominal lymph nodes**: No. Papers: n=0/19; No. Descriptions: n=0/23 | | | | | | | | | |
| 1 |  | |  | | |  | | | |
| **Lung**: No. Papers: n=4/15 No. Descriptions: n=10/24 | | | | | | | | | |
| 1 | Echo_cons_: hypo | n=4 | *Echo_cons_: hypo* | *Shape_cons_: round* | *n=3 (1x)* |  |  |  | *n=2 (4x)* |
| 2 | Shape_cons_: round | n=4 |  | | |  | | | |
| 3 |  | *n=3 (1x)* |  |  |  |  |  |  |  |
| **Intestines**: No. Papers: n=1/14; No. Descriptions: n=5/21 | | | | | | | | | |
| 1 | *Wall_thick_: thickening* | *n=3 (2x)* |  |  | *n=2 (3x)* | *n=2 (1x)* | | | |
| **Thyroid**: No. Papers: n=0/15; No. Descriptions: n=0/19 | | | | | | | | | |
| 1 |  | |  | | |  | | | |
| **Pleura**: No. Papers: n=0/12; No. Descriptions: n=0/18 | | | | | | | | | |
| 1 |  | |  | | |  | | | |
| **Prostate and seminal gland**: No. Papers: n=1/8; No. Descriptions: n=1/11 | | | | | | | | | |
| 1 |  | *n=1 (7x)* |  |  | *n=1 (21x)* |  |  |  | *n=1 (25x)* |
| **Bones**: No. Papers: n=1/10; No. Descriptions: n=1/12 | | | | | | | | | |
| 1 |  | *n=1 (5x)* | *Number_lesion_: single* | *Echo_lesion_: mixed* | *n=1 (10x)* |  |  |  | *n=1 (10x)* |
| **Myocardium**: No. Papers: n=1/9 No. Descriptions: n=1/9 | | | | | | | | | |
| 1 |  | *n=1 (5x)* |  |  | *n=1 (10x)* |  |  |  | *n=1 (10x)* |
| **Ovaries**: No. Papers: n=0/8; No. Descriptions: n=0/9 | | | | | | | | | |
| 1 |  | |  | | |  | | | |
| **Uterus**: No. Papers: n=0/6; No. Descriptions: n=0/8 | | | | | | | | | |
| 1 |  | |  | | |  | | | |
| **Eyes**: No. Papers: n=0/7; No. Descriptions: n=0/8 | | | | | | | | | |
| 1 |  | |  | | |  | | | |

**e) Exemplary results and discussion of selected organ descriptions**

Additional exemplary results for organ descriptions as derived from the organ feature ranking (Table S2)

*Liver*

The liver is the second most frequently described organ with 30 papers providing 40 descriptions (Table 2). Of 40 ultrasound pattern descriptions, the most common features extracted were [“hypoechoic echogenicity”] (24/40, 60%) and [“multiple number”] (21/40, 53%) and [“round/oval shape”] (17/40, 43%). The most common pairwise combinations of features were [“hypoechoic echogenicity” and “multiple number”] (14/40, 35%); [“hypoechoic echogenicity” and “round/oval shape”] (11/40, 28%); and [“disseminated location” and “multiple number”] of lesions (10/40, 25%). The most common feature triads were [“small size” and “hypoechoic echogenicity” and “multiple number”] and ["disseminated location” and “hypoechoic echogenicity” and “multiple number”] of lesions (8/40, 20% each).

*Spleen*

In our search, we identified 31 ultrasound pattern descriptions. The most common ultrasound features were [“hypoechoic echogenicity”] (24/31, 77%), [“multiple number”] (22/31, 71%),

[“small size”] (11/31, 35%) and [“well-defined margins”] of lesions (11/31, 35%). The most common pairwise combinations of features were [“hypoechoic echogenicity” and “multiple number”] of lesions (19/31, 61%); [“hypoechoic echogenicity” and “small size”] of lesions (10/31, 32%); [“small size” and “multiple number”] of lesions (10/31, 32%). The most common triad of features were [“hypoechoic echogenicity” and “small size” and “multiple number”] of lesions (9/31, 29%); [“hypoechoic echogenicity” and “multiple number” and “disseminated”] location of lesions in the spleen (7/31, 23%).

*Pericardium*

Pericardial TB was decribed in 21 publications detailing 26 descriptions. The most common features were ["large amount of effusion”] (15/26, 58%), ["effusion containing strands”] (12/26, 46%) and ["mixed echogenicity of effusion”] (9/26, 35%). The most common pairwise combination of features were ["large amount of effusion” and "effusion containing strands”] (9/26, 35%) and ["large amount of effusion” and "mixed echogenicity of effusion”] (8/26, 31%). The most common triad of features was ["large amount of effusion” and “mixed echogenicity of effusion” and “effusion containing strands”] (4/26, 15%).

*Abdominal lymph nodes (Supplement)*

Abdominal lymph nodes were described in 19 publications detailing 23 descriptions. The most common attributes were ["enlarged size”], ["multiple number”], ["hypoechoic echogenicity”] and ["rounded shape”] of lymph nodes. The most common 2tuples of attributes were ["enlarged size” and “hypoechoic echogenicity”] (10/23, 43%), ["enlarged size” and “multiple number”] (10/23, 43%) and ["enlarged size” and “rounded shape”] (7/23, 30%) of lymph nodes. The most common 3tuple of attributes were ["enlarged size” and “hypoechoic echogenicity” and “multiple number”], ["enlarged size” and “hypoechoic echogenicity” and “rounded shape”] and ["enlarged size” and “multiple number” and “rounded shape”] of lymph nodes (6/23, 26% each).

*Pleura (Supplement)*

Pleural changes were available in 18 descriptions coming from 12 publications. The most common attributes were ["presence of pleural fluid”] (16/18, 89%) and [“pleural fluid with stranding”] (9/18, 50%). The most common 2tuples were ["presence of pleural fluid” and “pleural fluid with stranding”] (9/18, 50%) and ["presence of pleural fluid” and “pleural fluid with mixed echogenicity”] (6/18, 33%). The most common 3tuple was ["presence of pleural fluid” and “pleural fluid with mixed echogenicity” and “pleural fluid with stranding”] (4/18, 22%).

*Lung*

We included 15 publications detailing 24 ultrasound descriptions and, the most common features were [“hypoechoic echogenicity”] (7/24, 29%) and [“multiple number”] (6/24, 25%). [“oval shape”], [“large size”], [“small size”], [“posterior enhancement”], [“pleural line irregularities”], [“B-lines”] and [“multiple lung zones affected”] were described in 5/24 (21%). The most common pairwise combinations of features were [“hypoechoic echogenicity” and “round shape”] of consolidations; [“pleural line irregularities” and “B-lines”]; [“pleural line irregularities” and “multiple lung zones affected”] (each with 4/24, 17%). The most common triad of features were [“hypoechoic echogenicity” and “round shape” and “small size”] of consolidations; [“hypoechoic echogenicity” and “round shape” and “posterior enhancement”] of consolidations; [“hypoechoic echogenicity” and “small size” and “posterior enhancement”] of consolidations; [“round shape” and “small size” and “posterior enhancement”] of consolidations; [“pleural line irregularities” and “B-lines” and “multiple lung zones affected”] with 3/24 (13%) each.

*Esophagus*

Other analyses showed associactions with neigbouring structures, as seen for esophageal TB. For esophageal TB we identified 26 ultrasound pattern descriptions. The most common single features were [“disrupted wall architecture”] (16/26, 62%), [“mixed echogenicity”] of wall lesions (13/26, 50%), [“continuity of lesions with mediastinal lymph nodes”] (12/26, 46%) and [“wall-thickening”] (10/26, 38%). The most common pairwise combinations of features were [“mixed echogenicity” and “multiple number”] of lesions and [“wall-thickening” and “disrupted wall architecture”] (7/26, 27% each). The most common triad of features were “mixed echogenicity” and “multiple number” and “well-defined margin”] of lesions (6/26, 23%) and [“mixed echogenicity” and “single number” of lesions and “disrupted wall architecture”] (5/26, 19%). Any mediastinal lymph nodes were described in 17/26 (65%) quotations (with continuity to esophagus n=12, no continuity mentioned n=5).

Additional discussion regarding the supplemental results above

Regarding the FASH protocol, which has been subject to various studies in different populations and our review mirrors the findings that were included in that protocol (1, 2): The TB ultrasound pattern of the liver is dominated by multiple, small, and hypoechoic lesions suggesting disseminated or miliary TB and is also the pattern that can be derived for the spleen. A rarer spleen pattern was cases with single, well-defined lesions, but only 6/31, 19% had this pair. The ultrasound features for pericardial effusion add to the binary reporting of the FASH protocol (effusion present or not?) by adding distinct sonographic features frequently described in TB to possibly differentiate it from other etiologies (i.e., large amount, stranding, mixed echogenicity). While in part repeating protocol items of FASH, our review highlights in greater depth the features that are found in FASH-organs.

The field of lung ultrasound received broader attention in infectious disease research in the early SARS-CoV-2 pandemic (3) and limited evidence from TB-related studies have discussed LUS for TB (4). Of 15 publications included in our analysis, 11 have been published in 2016 or later. The features cluster in two patterns: one with round hypoechoic consolidations with posterior enhancement and another best described as a generalized pleural pattern with irregularities and B-lines (cf., Table 2, 3). These reflect very well the patterns “subpleural nodules” and “miliary pattern” from different publications (5, 6) and summarized in a systematic review (4), confirming the adequacy of our tupel-ranking approach.

Beyond these more deeply investigated organs, our review revealed hints of associations between TB manifestations, as is the case for the exemplary organ esophagus: The clustering of features around esophageal wall lesions and the association with mediastinal lymphadenopathy led us to the summary “esophageal wall architecture disruptions with thickening and mixed-echogenicity lesions which are often contiguous with surrounding lymph nodes”. This association has also been hypothesized in the literature (7, 8) and may be a hint to pathogenesis (e.g., primary lymph node TB infiltrating the esophagus or primary esophagus TB seeding into nearby lymph nodes).

REFERENCES

1. Heller T, Wallrauch C, Goblirsch S, Brunetti E. Focused assessment with sonography for HIV-associated tuberculosis (FASH): a short protocol and a pictorial review. Critical ultrasound journal. 2012;4(1):21-.

2. Van Hoving DJ, Griesel R, Meintjes G, Takwoingi Y, Maartens G, Ochodo EA. Abdominal ultrasound for diagnosing abdominal tuberculosis or disseminated tuberculosis with abdominal involvement in HIV-positive individuals. Cochrane Database Syst Rev. 2019;9:Cd012777.

3. Smith MJ, Hayward SA, Innes SM, Miller ASC. Point-of-care lung ultrasound in patients with COVID-19 - a narrative review. Anaesthesia. 2020;75(8):1096-104.

4. Bigio J, Kohli M, Klinton JS, MacLean E, Gore G, Small PM, et al. Diagnostic accuracy of point-of-care ultrasound for pulmonary tuberculosis: A systematic review. PLoS One. 2021;16(5):e0251236.

5. Agostinis P, Copetti R, Lapini L, Badona Monteiro G, N'Deque A, Baritussio A. Chest ultrasound findings in pulmonary tuberculosis. Trop Doct. 2017;47(4):320-8.

6. Montuori M, Casella F, Casazza G, Franzetti F, Pini P, Invernizzi C, et al. Lung ultrasonography in pulmonary tuberculosis: A pilot study on diagnostic accuracy in a high-risk population. Eur J Intern Med. 2019;66:29-34.

7. Maulahela H, Fauzi A, Renaldi K, Srisantoso QP, Jasmine A. Current role of endoscopic ultrasound for gastrointestinal and abdominal tuberculosis. JGH Open. 2022;6(11):745-53.

8. Abu-Zidan FM, Sheek-Hussein M. Diagnosis of abdominal tuberculosis: lessons learned over 30 years: pectoral assay. World J Emerg Surg. 2019;14:33.
