# Supplementary material for: Tuberculosis Visualized With the Ultrasound Probe: A Systematic Review of Sonographic Pattern Descriptions and an Analysis of Common Sonographic Features
Source: Open Forum Infect Dis. 2025 Mar 7;12(3):ofaf010. doi: 10.1093/ofid/ofaf010 (PMC11886843; doi:10.1093/ofid/ofaf010)
Supplement: ofaf010_Supplementary_Data [file ofaf010_supplementary_data.zip › 3 S2 Search protocol_R1_clean.docx]

# Systematic literature search

## Purchaser

Herr Stefan Weber

Tropenambulanz der UK Heidelberg

Assigned 03.08.2021

Delivered 30.08.2021

## Executed by

Maurizio Grilli M.L.I.S.

Professional Information Biomedicine and Health Professions

Roonstr. 17

76137 Karlsruhe

Fixed line: +49 (0)721 / 9766 1750

Mobile: +49 (0)1577 / 7823208

| **Current status** |  | **Number of references** |  | **Next action** |
| --- | --- | --- | --- | --- |
| After deletion of duplicates | 🡪 | 4314 | 🡪 | Remove all <2000 and wrong language |
| After removal <2000 and wrong language | 🡪 | 3374 | 🡪 | 1^st^ screening (title, abstract) |
| Title and abstract screening remaining | 🡪 | 1389  1172 english  62 french  37 german  5 italian  17 spanish  96 unclear | 🡪 | Full text screening |
| After full text screening | 🡪 | 380 included  920 excluded  5 Duplicates 🡪 excluded  8 Wrong language in full text 🡪 excluded  76 no full text available (despite author contact) 🡪 excluded | 🡪 | Additional refs from other sources (SR) |
| 8 papers added | 🡪 | **Total count 388** |  |  |

## Topic

What evidence exists for ultrasound in the diagnosis of tuberculosis?

## Main topic concepts definition

### P

| tuberculosis |  |
| --- | --- |

### I

| ultrasound |  |
| --- | --- |

### O

| diagnosis |  |
| --- | --- |

## Strategy

| 1 | P |  |
| --- | --- | --- |
| 2 | I |  |
| 3 | O |  |
| 4 | 1 AND 2 AND 3 |  |

## Databases

- PubMed
- Cochrane Library
- Web of Science Core Collection
- Cinahl
- Clinical Trials.Gov
- WHO ICTRP

## Results report

The results were saved in Endnote and deduplicated. Some articles could still appear more than once.

The hits are sorted by database in Endnote. The PubMed hits were the first to be exported in Endnote. This makes them preferred for deduplication. In other words, in the case of duplicates, entries are removed from other databases.

The number of hits for each database in this report is based on its pre-deduplication status in EndNote.

## PubMed

| **Records number** | **Date** |
| --- | --- |
| 3873 | 30.08.2021 |

### Search Terms

### P

| **"Tuberculosis"[Mesh] OR**  Tuberculo*[tiab] OR  TB[tiab] | 286450 |
| --- | --- |

### I

| **"Ultrasonography"[Mesh] OR**  **"Echocardiography"[Mesh] OR**  **"Point-of-Care Systems"[Mesh] OR**  “Point of Care”[tiab] OR  Ultrasound*[tiab] OR  Ultra sound*[tiab] OR  Ultrasonic*[tiab] OR  Ultra sonic*[tiab] OR  Sonograph*[tiab] OR  Sono graph*[tiab] OR  Sonogram*[tiab] OR  Ultrasonogr*[tiab] OR  Echotomography[tiab] OR  Echo tomograph*[tiab] OR  echocardiograph*[tiab] OR  echo cardiograph*[tiab] | 756451 |
| --- | --- |

### O

| **"Diagnosis"[Mesh] OR**  Diagnos*[tiab] | 10270491 |
| --- | --- |

## Cochrane Library

| **Records number** | **Date** |
| --- | --- |
| 184 | 30.08.2021 |

### P

| [mh "Tuberculosis"] OR  (Tuberculo*):ti,ab,kw OR  (TB):ti,ab,kw | 8323 |
| --- | --- |

### I

| [mh "Ultrasonography"] OR  [mh "Echocardiography"] OR  [mh "Point-of-Care Systems"] OR  (Point NEAR/3 of NEAR/3 Care):ti,ab,kw OR  (Ultrasound*):ti,ab,kw OR  (Ultra NEAR/3 sound*):ti,ab,kw OR  (Ultrasonic*):ti,ab,kw OR  (Ultra NEAR/3 sonic*):ti,ab,kw OR  (Sonograph*):ti,ab,kw OR  (Sono NEAR/3 graph*):ti,ab,kw OR  (Sonogram*):ti,ab,kw OR  (Ultrasonogr*):ti,ab,kw OR  (Echotomography):ti,ab,kw OR  (Echo NEAR/3 tomograph*):ti,ab,kw OR  (echocardiograph*):ti,ab,kw OR  (echo NEAR/3 cardiograph*):ti,ab,kw | 61589 |
| --- | --- |

### O

| [mh "Diagnosis"] OR  (Diagnos*):ti,ab,kw | 500637 |
| --- | --- |

## Search strings

(as in the table above)

## Web of Science Core Collection

| **Records number** | **Date** |
| --- | --- |
| 1572 | 30.08.2021 |

### P

| "Tuberculo*" OR  "TB" | 197,906 |
| --- | --- |

### I

| "Point of Care" OR  "Ultrasound*" OR  "Ultra sound*" OR  "Ultrasonic*" OR  "Ultra sonic*" OR  "Sonograph*" OR  "Sono graph*" OR  "Sonogram*" OR  "Ultrasonogr*" OR  "Echotomography" OR  "Echo tomograph*" OR  "echocardiograph*" OR  "echo cardiograph*" | 622,888 |
| --- | --- |

### O

| "Diagnos*" | 2,211,857 |
| --- | --- |

The search was conducted in the fields title and abstract.

## CINAHL

| **Records number** | **Date** |
| --- | --- |
| 732 | 30.08.2021 |

### P

| "Tuberculo*" OR  "TB" | 35666 |
| --- | --- |

### I

| "Point of Care" OR  "Ultrasound*" OR  "Ultra sound*" OR  "Ultrasonic*" OR  "Ultra sonic*" OR  "Sonograph*" OR  "Sono graph*" OR  "Sonogram*" OR  "Ultrasonogr*" OR  "Echotomography" OR  "Echo tomograph*" OR  "echocardiograph*" OR  "echo cardiograph*" | 188838 |
| --- | --- |

### O

| "Diagnos*" | 1123194 |
| --- | --- |

## Clinical Trial Gov

<http://www.clinicaltrials.gov/>

| **Records number** | **Date** |
| --- | --- |
| 89 | 30.08.2021 |

### P

| Tuberculosis OR  TB |  |
| --- | --- |

### I

| "Point of Care" OR  Ultrasound OR  Ultrasonic OR  Sonography OR  Sonogram OR  Ultrasonography OR  Echotomography OR  echocardiography |  |
| --- | --- |

### O

| Diagnosis |  |
| --- | --- |

### Search strings

|  |  | **Records number** |
| --- | --- | --- |
| **P** | (Tuberculosis OR TB)  AND | 2001 |
| **I** | ("Point of Care" OR Ultrasound OR Ultrasonic OR Sonography OR Sonogram OR Ultrasonography OR Echotomography OR echocardiography)  AND | 23260 |
| **O** | Diagnosis | 64212 |

## International Clinical Trials Registry Platform ICTRP (WHO Trials)

<https://trialsearch.who.int/Default.aspx>

| **Records number** | **Date** |
| --- | --- |
| 5 | 30.08.2021 |

### P

| Tuberculosis OR  TB |  |
| --- | --- |

### I

| Point of Care OR  Ultrasound OR  Ultrasonic OR  Sonography OR  Sonogram OR  Ultrasonography OR  Echotomography OR  echocardiography |  |
| --- | --- |

### O

| Diagnosis |  |
| --- | --- |

## Search strings

|  | **Search strings** | **Records number** |
| --- | --- | --- |
| **Title** | Diagnosis | 6976 |
| **Condition** | Tuberculosis OR TB | 2033 |
| **Intervention** | Point of Care OR Ultrasound OR Ultrasonic OR Sonography OR Sonogram OR Ultrasonography OR Echotomography OR echocardiography | 1093 |
